# Single-cell transcriptome profiling reveals the spatiotemporal distribution of triterpenoid saponin biosynthesis and transposable element activity in *Gynostemma pentaphyllum* shoot apexes and leaves

**DOI:** 10.3389/fpls.2024.1394587

**Published:** 2024-05-08

**Authors:** Rucan Li, Ke Du, Chuyi Zhang, Xiaofeng Shen, Lingling Yun, Shu Wang, Ziqin Li, Zhiying Sun, Jianhe Wei, Ying Li, Baolin Guo, Chao Sun

**Affiliations:** ^1^ Institute of Medicinal Plant Development, Chinese Academy of Medical Sciences and Peking Union Medical College, Beijing, China; ^2^ College of Pharmacy, Shandong University of Traditional Chinese Medicine, Jinan, Shandong, China

**Keywords:** single-cell, RNA-seq, *Gynostemma pentaphyllum*, triterpenoid saponin, transposable element

## Abstract

*Gynostemma pentaphyllum* (Thunb.) Makino is an important producer of dammarene-type triterpenoid saponins. These saponins (gypenosides) exhibit diverse pharmacological benefits such as anticancer, antidiabetic, and immunomodulatory effects, and have major potential in the pharmaceutical and health care industries. Here, we employed single-cell RNA sequencing (scRNA-seq) to profile the transcriptomes of more than 50,000 cells derived from *G. pentaphyllum* shoot apexes and leaves. Following cell clustering and annotation, we identified five major cell types in shoot apexes and four in leaves. Each cell type displayed substantial transcriptomic heterogeneity both within and between tissues. Examining gene expression patterns across various cell types revealed that gypenoside biosynthesis predominantly occurred in mesophyll cells, with heightened activity observed in shoot apexes compared to leaves. Furthermore, we explored the impact of transposable elements (TEs) on *G. pentaphyllum* transcriptomic landscapes. Our findings the highlighted the unbalanced expression of certain TE families across different cell types in shoot apexes and leaves, marking the first investigation of TE expression at the single-cell level in plants. Additionally, we observed dynamic expression of genes involved in gypenoside biosynthesis and specific TE families during epidermal and vascular cell development. The involvement of TE expression in regulating cell differentiation and gypenoside biosynthesis warrant further exploration. Overall, this study not only provides new insights into the spatiotemporal organization of gypenoside biosynthesis and TE activity in *G. pentaphyllum* shoot apexes and leaves but also offers valuable cellular and genetic resources for a deeper understanding of developmental and physiological processes at single-cell resolution in this species.

## 1 Introduction

*Gynostemma pentaphyllum* (Thunb.) Makino is a perennial vine native to East Asia within the Cucurbitaceae family (Zhang et al., 2023). For centuries, this plant has been extensively used as a dietary herbal medicine due to its diverse pharmacological benefits, including antitumor, antiaging, immunomodulatory, and neuroprotective activities (Wang et al., 2020). Currently, *G. pentaphyllum* is readily available in various commercial formulations, such as tea, tablets, capsules, and powders (Niu et al., 2013). Additionally, extracts of *G. pentaphyllum* are utilized as additives in a range of products, including beverages, biscuits, bread, and noodles (Ji et al., 2018; Su et al., 2021). The major bioactive constituents of *G. pentaphyllum* are saponins, specifically gypenosides, the majority of which exist as dammarane-type triterpenoid saponins (Nguyen et al., 2021). More than 200 gypenosides have been identified in *G. pentaphyllum* (Nguyen et al., 2021). Notably, some gypenosides share identical structures with ginsenosides from *Panax ginseng*, making *G. pentaphyllum* the first ginsenoside-producing plant outside of the Araliaceae family (Zhang et al., 2023). Consequently, *G. pentaphyllum* may serve as an alternative resource for ginsenosides. The biosynthetic pathway of dammarane-type saponins in the genus *Gynostemma* has been partially elucidated. For instance, the first committed enzyme in this pathway is dammarenediol-II synthase (DS), which catalyzes the cyclization of 2,3-oxidosqualene to dammarenediol-II. A DS has been characterized from *G. longipes* (Ye et al., 2022). Furthermore, five UDP-dependent glycosyltransferases (UGTs), which are responsible for catalyzing the final steps of gypenoside biosynthesis, have been identified in *G. pentaphyllum* (Le et al., 2021). However, while cytochrome P450s (CYP450s) are predicted to play crucial roles in multiple hydroxylation steps, these processes have not been fully elucidated.

Single-cell RNA sequencing (scRNA-seq) technology offers an unparalleled opportunity to investigate intricate cellular and molecular processes by improving the spatiotemporal resolution of transcriptomic analysis to the level of the individual cell (Seyfferth et al., 2021). This cutting-edge technology has been extensively employed for the identification of rare and novel cellular entities, as well as for the elucidation of cellular differentiation and development in plant biology (Lopez-Anido et al., 2021). Notably, the use of scRNA-seq has been expanded to enhance the understanding of plant specialized metabolism. Compared to bulk RNA sequencing, scRNA-seq offers several advantages. These include providing single-cell resolution for gene expression profiling to resolve cellular heterogeneity, elucidating cell states and trajectories relevant to developmental processes, and revealing the spatiotemporal distribution of specialized metabolic pathways. For instance, scRNA-seq was used to dissect the spatial distribution of the vinblastine biosynthetic pathway in *Catharanthus roseus* leaves, and the pathway was found to be compartmentalized into three cell types: starting in internal phloem-associated parenchyma (IPAP) cells, followed by the intermediate enzymatic steps predominantly occurring in epidermal cells (ECs), and concluding with the late steps in idioblast cells (ICs) (Li et al., 2023; Sun et al., 2023). These findings are consistent with prior reports using RNA *in situ* hybridization. The integration of scRNA-seq with mass spectrometry imaging technology can further enhance our ability to explore the spatial organization of specialized metabolism. A combination of two technologies revealed that the majority of taxol biosynthesis genes are predominantly expressed in leaf mesophyll cells (MCs), while phenolic acid and flavonoid biosynthesis genes are highly expressed in leaf ECs (Zhan et al., 2023). In addition, a single-cell transcriptome atlas of tea leaves was constructed and a novel catechin ester glycosyltransferase was characterized by a gene coexpression network in MCs, suggesting that scRNA-seq has great potential for the screening and identifying genes involved in the biosynthesis of plant specialized metabolites (Wang et al., 2022).

Transposable elements (TEs) are DNA fragments characterized by their ability to mobilize or replicate within a host genome. In plants, in addition to their intrinsic transcription, TE transcripts strongly shape transcriptomic profiles by regulating host gene expression and chromatin accessibility (Fueyo et al., 2022). Given the diverse roles of TE transcripts in molding the host transcriptome, several computational tools have been developed to precisely quantify TE expression (Lanciano and Cristofari, 2020). Notably, two pipelines, scTE and soloTE, have been utilized to investigate TE expression at the single-cell level. For instance, the analysis of TE expression in mouse embryonic stem cells and during human cardiac differentiation using scTE revealed that specific TE types are expressed in subpopulations of embryonic stem cells and undergo dynamic regulation during pluripotency reprogramming, differentiation, and embryogenesis (He et al., 2021). Furthermore, soloTE was employed to determine the impact of TE expression on the cellular heterogeneity of early gastric cancer (Rodriguez-Quiroz and Valdebenito-Maturana, 2022). This investigation revealed that two TEs, L1PA7 and THE1D, exhibit higher expression in the cancer cells than in other cell types. Although investigations of TE expression have expanded to single-cell resolution in various animal systems, characterization of TE transcriptional dynamics in plants has previously been limited to tissue-level profiles.

Here, we constructed high-resolution single-cell transcriptome atlases of *G. pentaphyllum* shoot apexes and leaves. Most of the genes involved in the gypenoside biosynthetic pathway exhibited high expression in MCs, indicating that MCs serve as the primary site for gypenoside biosynthesis. Furthermore, these pathway genes exhibited distinct expression patterns during EC and VC development. Notably, this study marks the first exploration of TE expression at the single-cell resolution in the plant kingdom. TE activity exhibited an uneven distribution among different cell types and during cell differentiation and development. Overall, this work provides novel insights into the spatiotemporal organization of triterpenoid saponin biosynthesis and TE activity in *G. pentaphyllum* shoot apexes and leaves. Additionally, the abundant datasets generated herein also establish a foundation for further elucidating developmental and physiological processes at single-cell resolution in this species.

## 2 Materials and methods

### 2.1 Plant material and protoplast preparation

*G. pentaphyllum* (Thunb.) Makino plants were grown at 25°C in a greenhouse under a 16/8 h photoperiod. Leaves and shoot apexes of young healthy stolons were harvested for protoplasting. Two biological replicates were prepared for each tissue. Protoplasts were obtained by enzymatic hydrolysis. Approximately 0.15 g of tender leaves and shoot apexes of *G. pentaphyllum* were harvested and cut into small pieces. The samples were treated with enzymolysis solution (1% cellulase R-10, 0.15% macerozyme R-10, 0.1% pectinase Y‐23, 0.45 M mannitol and 20 mM MES, pH 5.7 - 5.8). The samples were incubated in darkness for 3.0 - 3.5 h at 25°C to isolate the protoplasts. After reaching a certain number of protoplasts were obtained and passed through 70-µm and twice 40-µm strainers, the protoplasts were centrifuged at 100 × g for 5 min and washed once with protoplasting solution (0.45 M mannitol and 20 mM MES, pH 5.7 - 5.8) without enzymes. Protoplasts were placed on ice until further processing. Protoplasts were stained with trypan blue (0.2% final) and checked on a hematocytometer under a Leica M205FA microscope to determine cell viability and concentration. The final cell concentration was adjusted to a range from 1,500 to 1,800 cells µl^−1^.

### 2.2 scRNA-seq library construction and sequencing

The scRNA-seq libraries were constructed using the Chromium Single-cell 3′ Gel Beads-in-emulsion (GEM) Library and Gel Bead Kit v.3 (16 rxns PN-1000268, 10x Genomics) according to the user’s manual supplied with the kit. In this study, approximately 20000 cells were counted per sample. The concentration of the DNA library was measured by a Qubit3.0. Qualitative analysis of the DNA library was performed with an Agilent 2100 Bioanalyzer. Libraries were sequenced on an Illumina HiSeq 4000 according to the manufacturer’s instructions.

### 2.3 Preprocessing of scRNA-seq data

The *G. pentaphyllum* telomere-to-telomere (T2T) genome was downloaded from the National Center for Biotechnology Information (NCBI) (BioProject: PRJNA1030183). The raw reads generated via high-throughput sequencing were in the FASTQ format. The Alevin pipeline (Salmon v.0.6.0) (Srivastava et al., 2019) was used to count the unique molecular identifiers (UMIs) and construct digital expression matrices, using the command ‘salmon alevin’ with the arguments ‘expectCells=15000’. We loaded the matrices of two leaf and two shoot apex to create seurat objects using the R package Seurat (v.4.3.1) (Hao et al., 2021). To obtain quality control statistics, such as high-quality cell numbers, gene medians, and sequencing saturation, we first specified the quality control standard, Cells with <500 and >6,500 detected genes, UMI counts <500 and >25,000, mitochondrial reads with percentages >3% and chloroplast genes with percentages >40% were removed. Doublets were filtered out by DoubletFinder (v.2.0.3) (McGinnis et al., 2019) with default settings. The cell cycle score was calculated using the CellCycleScoring function in Seurat and using the genes in **Supplementary Table 12**. We then used regularized negative binomial regression to normalize UMI counts using the SCTransform (vst.flavor = “v2”) function in Seurat, with the percentage of mitochondrial genes, chloroplast genes, UMI counts and the cell cycle score regressed out. Principal component analysis (PCA) was performed using the RunPCA function based on highly variable genes detected using the SelectIntegrationFeatures function and 3,000 features. Batch correction was eliminated with the RunHarmony function using the R package Harmony (v.0.1.1) (Korsunsky et al., 2019), with the assay (parameter’assay.use’) set as ‘SCT’.

### 2.4 Cell clustering and annotation

We performed nearest-neighbor graph construction using the FindNeighbors function with the ‘reduction’ parameter in the FindNeighbors set as ‘harmony’, ‘dims=20’. The UMAP algorithm was used to perform nonlinear dimensionality reduction and visualization of all cells with the RunUMAP function, with parameter ‘dims=20’. We determined graph-based clustering using the FindClusters function, and used clustree package (v.0.5.1) to select the appropriate resolution by plotting the clustering results at 0-1 resolution. The different clusters were extracted using the FindAllMarkers function, setting pct.2 < 0.1 and logfc.threshold=0.25 as thresholds to identify cell type-specific genes in particular clusters. After that, we selected upregulated cell type-specific genes in different clusters for GO enrichment analysis using ClusterProfiler (v.3.18.1). The simultaneous visualization of cell distribution and marker gene expression in two-dimensional space was performed using the Feature plot function.

### 2.5 Pseudotime analysis

Pseudotime trajectory analysis was conducted utilizing the Monocle R package (v.2.8.0) (Qiu et al., 2017). The log-normalized data derived from the Seurat object were imported into Monocle through the application of the as.CellDataSet function. Subsequently, the cells were ordered along the trajectory and presented in a reduced dimensional space. The determination of the trajectory root was performed according to the cell subcluster identities. To discern genes exhibiting significant changes along pseudotime, the differentialGeneTest function was applied, with the stringent criterion of a q value < 0.1. The identified genes were subsequently subjected to clustering using the plot_pseudotime_heatmap function, adhering to default parameters to ensure robust and reliable outcomes. Specifically, genes demonstrating dynamic expression patterns throughout pseudotime were visually represented through the plot_genes_in_pseudotime function, enhancing the granularity of our analysis. Differentially expressed genes including cluster-enriched genes with statistical significance were submitted to TBtools for GO enrichment analysis (Chen et al., 2020). The top 30 GO terms with -log_10_p-values were represented.

### 2.6 Analysis of scRNA-seq data using scTE

The TEs of the reference genome were annotated by RepeatModeler (v2.0.5) (Flynn et al., 2020), TEclass (v2.1.3C) (Abrusán et al., 2009) and RepeatMasker (v4.1.5). The annotation files of the genes and TEs were used to determine indices through the commander ‘scTE_build’ of the scTE (v1.0.4) (He et al., 2021) pipeline. The BAM file required for the pipeline was quantitatively generated by CellRanger (v7.0.1) from the single cell data. Consequently, the gene/TE expression matrix was generated through the commander ‘scTE’ with the arguments ‘-x, -i, -o, –expect-cells’ and was used in Seurat (v4.3.1) for further analysis via the same process as that used for the analysis based on gene expression alone.

### 2.7 RNA *in situ* hybridization

For *in situ* hybridization assays, leaves harvested during the same growth phase as those used for protoplast isolation were fixed with formaldehyde-acetic acid-ethanol fixative (50%) at 4°C for 24 h, followed by manual embedding. The unique fragments of selected genes were cloned into the pGEM-T easy vector (Promega, catalogue no. A1360). The primers used are listed in **Supplementary Table 15**. For RNA probe synthesis, linearized vectors were added as templates. And the subsequent *in vitro* transcription was performed with a DIG RNA Labeling Kit (Sp6/T7) (Roche, catalogue no. 11175025910). The leaves were paraffin-embedded and sectioned (10 μm) with a sliding microtome (Leica). The sections were then affixed onto slides coated with 3-aminopropyltriethoxysilane (AES)-coated slides (WHITE 12-550-15, Thermo Fisher Scientific) overnight at 42 °C, and paraffin was removed using xylene (twice for 15 min) before rehydration in an ethanol gradient up to diethypyrocarbonate (DEPC)-treated water. Following rehydration, the sections were subjected to Proteinase K (Sigma, catalogue no. P2308) digestion, dehydrated via a series of ethanol solutions and hybridized with RNA probes. After washing, sections were incubated with anti-digoxigenin-AP Fab fragments (Roche, catalogue no. 11093274910). For color development, sections were immersed in NBT/BCIP (Sigma, catalogue no. B5655) staining solution at room temperature until the target color was clear. Microscopy was carried out in bright-field mode using BioTek CYTATION/5 imaging reader.

## 3 Results

### 3.1 Construction of the *G. pentaphyllum* shoot apex and leaf cell atlases

**Figure 1:**
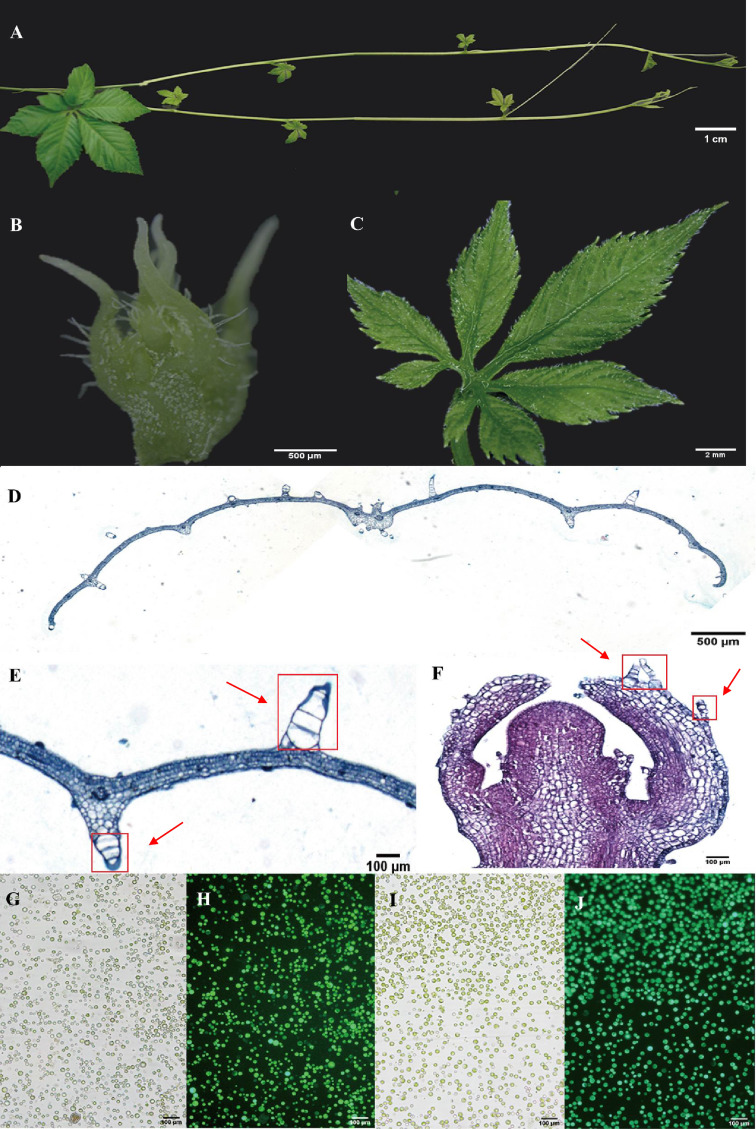
Anatomical features and protoplasts of *G. pentaphyllum* shoot apexes and leaves. **(A)** The stolon of *G. pentaphyllum*. **(B, C)** Shoot apex and young leaf for protoplast preparation and tissue section staining. **(D-F)** Leaf cross-sections and shoot apex longitudinal sections stained with Safranin-O and Fast Green. The red boxes represent the trichomes. **(G, I)** Protoplasts of shoot apexes and leaves under bright light. **(H, J)** Protoplasts of shoot apexes and leaves stained with fluorescein diacetate. Live cells can emit green fluorescence (488 nm) after staining with fluorescein diacetate.

Leaves and young stems serve as prominent tissues for gypenoside biosynthesis in *G. pentaphyllum* (**Figures 1A–C**). Anatomical analyses were conducted using light microscopy on both leaf cross-sections and longitudinal sections of shoot apexes. After staining with Safranin-O and Fast Green, the sections were observed under a visible light microscope. Leaf sections revealed distinct upper and lower epidermal cell layers surrounding mesophyll parenchyma tissue and vascular bundles. Both the adaxial and abaxial epidermal tissues exhibited abundant large trichome structures (**Figures 1D, E**). In contrast, shoot apex tissues predominantly consisted of saffron-stained susceptible parenchymal tissue along with epidermal cell layers bearing smaller trichomes than did leaf tissues (**Figures 1F**). Protocols for protoplast isolation and purification were optimized, yielding highly viable protoplasts derived from vegetative shoot apexes and young leaves (**Figures 1G–J**). These intact protoplasts from specific gypenoside biosynthetic tissues were subsequently used to generate single-cell transcriptomic datasets.

**Figure 2:**
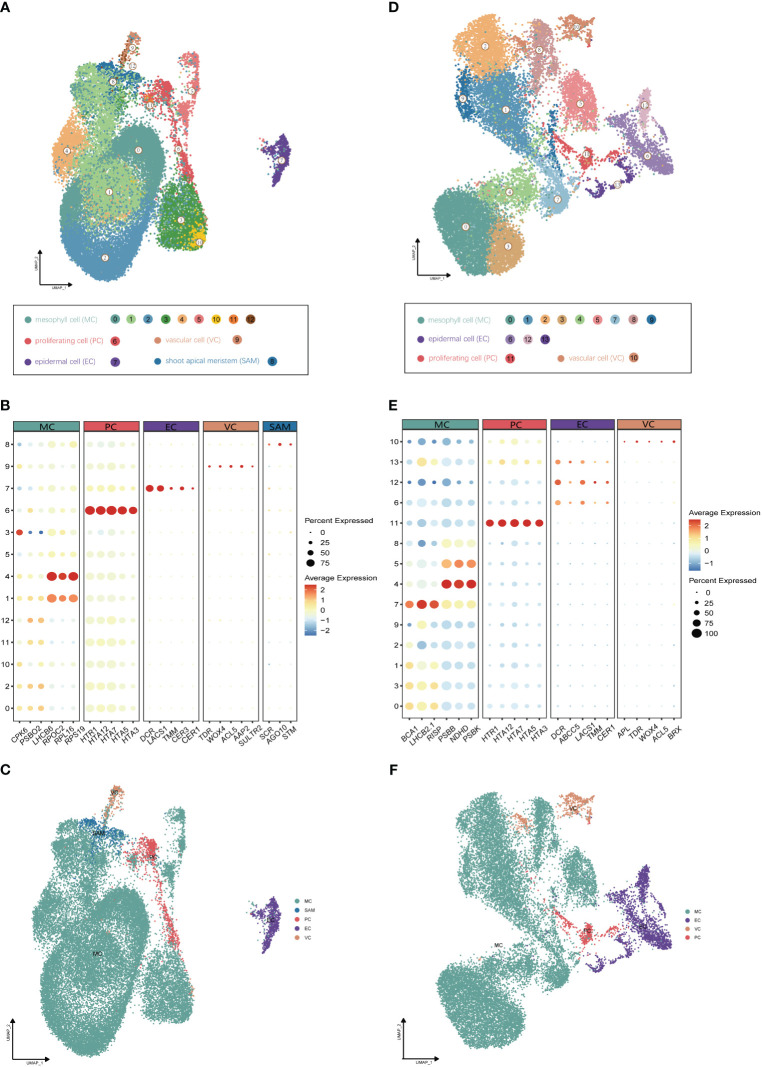
Identification of cell clusters from (G) *pentaphyllum* shoot apexes and leaves. **(A)** UMAP visualization of 13 cell clusters in shoot apexes. Each dot represents an individual cell; the total number of cells (n) is 29,395. Colors indicate different cell clusters. **(B)** Expression patterns of representative cluster-specific marker genes in shoot apexes. Dot diameter represents the proportion of cluster cells expressing a given gene. Full names of the selected genes are provided in **Supplementary Table 3**. **(C)** UMAP visualization of five broad populations of shoot apexes. Colors represent different population types. **(D)** UMAP visualization of 14 cell clusters in leaves. Each dot represents an individual cell; the total number of cells (n) is 21,103. Colors indicate different cell clusters. **(E)** Expression patterns of representative cluster-specific marker genes in leaves. Dot diameter represents the proportion of cluster cells expressing a given gene. Full names of the selected genes are provided in **Supplementary Table 3**. **(F)** UMAP visualization of four broad populations in leaves. Colors represent different population types.

Utilizing Illumina high-throughput sequencing, a total of 580 Gb of scRNA-seq data were generated from four distinct single-cell complementary DNA libraries originating from young leaf protoplasts and vegetative shoot apex protoplasts of *G. pentaphyllum*, with two biological replicates for each tissue (**Supplementary Table 1**). To ensure accurate quantification of the single-cell transcriptome, we utilized the reference-level telomere-to-telomere (T2T) genome of *G. pentaphyllum* (BioProject: PRJNA1030183). Following the acquisition of scRNA-seq datasets, Alevin was employed for quantification. After filtering, a high-quality dataset, comprising 21,103 and 29,395 cells from shoot apexes and leaves, respectively, was obtained. In the leaves, the median number of genes per cell was 2,249 and the median number of UMIs per cell was 4,510; in the shoot apexes, the median number of genes and UMIs per cell were 2,435 and 4,849, respectively (**Supplementary Table 2**). For each tissue, principal component analysis (PCA) of the 3000 most highly variable genes were performed for the dimensional reduction of the scRNA-seq data. Unsupervised clustering identified of 13 and 14 distinct cell clusters in shoot apexes and leaves, respectively. These clusters were visualized using two-dimensional uniform manifold approximation and projection (UMAP) plots (**Figure 2**).

**Figure 3:**
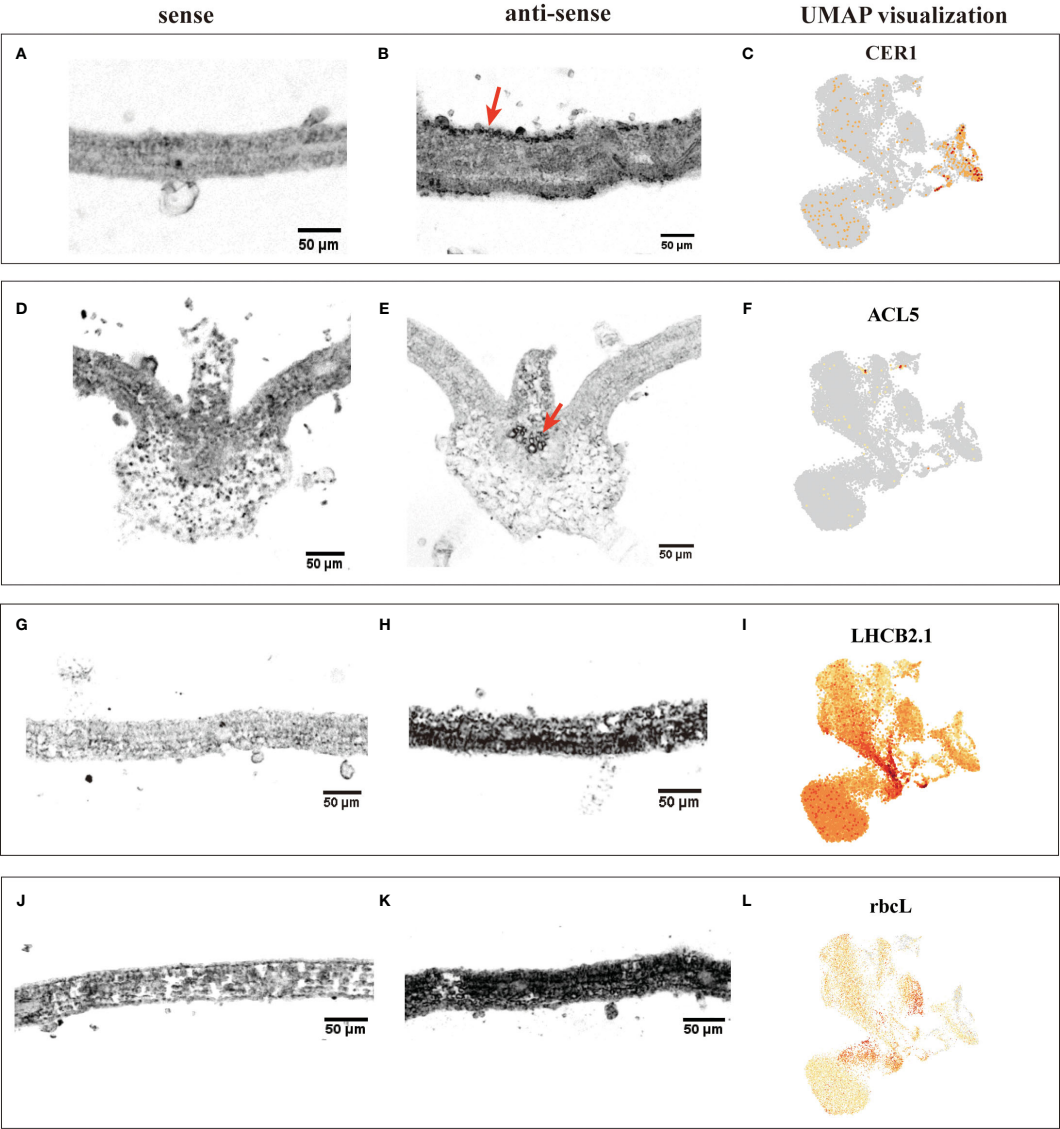
RIH validating the annotated cell identities in *G. pentaphyllum* leaves and UMAP visualization of the transcript accumulation of cell type-specific marker genes. **(A-C)** EC (*CER1*); **(D-F)**, VC (*ACL5*); **(G-I)**, MC (*LHCB2.1*); **(J-K)**, MC (*rbcL*). In the hybridized sections, the sense was the control, the black indicated the location of the signal in the anti-sense, the red arrows were the hybridization signal of ECs and VCs. On the UMAP plot, the color intensity represents the relative transcript expression level for the indicated gene in each cell.

The annotation of cell clusters was based on cell-type markers, whose functions have been well characterized in *Arabidopsis* (**Supplementary Table 3**). To ensure annotation accuracy, more than three cell-type markers were utilized for each cell type in *G. pentaphyllum* shoot apexes and leaves. For the shoot apexes, the gene markers specific to MCs, including Oxygen-evolving enhancer protein 1-2 (*PSBO2*, Gynpe.TU.chr03.1614) and Chlorophyll a-b binding protein (*LHCB6*, Gynpe.TU.chr10.262), exhibited a concentrated expression pattern within Clusters 0-5 and 10-12 (**Figures 2A–C**) (Murakami et al., 2005; Chen et al., 2018). These clusters collectively delineated the population identified as MCs. Similarly, epidermal cell-specific genes, including *TOO MANY MOUTHS* (*TMM*, Gynpe.TU.chr04.1612) and *ECERIFERUM1* (*CER 1*, Gynpe.TU.chr06.3193) (Bhave et al., 2009; Bernard et al., 2012), were prominently expressed in Cluster 7, which was designated as epidermal cells (ECs) (**Figure 2B**). Cluster 9 was annotated as vascular cells (VCs), in which genes involved in governing vascular procambium differentiation and xylem specification, such as *WUSCHEL-related homeobox 4* (*WOX4*, Gynpe.TU.chr06.1767) and thermospermine synthase ACAULIS5 (*ACL5*, Gynpe.TU.chr06.1882), were predominantly expressed (**Figure 2B**) (Muniz et al., 2008; Ji et al., 2010). Moreover, Cluster 6 was designated as proliferating cells (PCs) due to the enrichment of genes associated with histone function, exemplified by histone H2A.Z (*HTA3*) (**Figure 2B**). Finally, Cluster 8 was designated as shoot apical meristem cells (SAMs) because *SCARECROW* (*SCR*, Gynpe.TU.chr07.931) and proteins such as the protein argonaute 10 (*AGO10*, Gynpe.TU.chr02.1132), which is essential for stem cell function, were markedly overrepresented in this cluster (**Figure 2B**) (Sabatini et al., 2003; Liu et al., 2009). In the leaves, the same method was used to annotate cell types. Finally, Clusters 0-5 and 7-9 were collectively identified as mesophyll cells (MCs), while Clusters 6, 12, and 13 were designated as ECs, and Clusters 10 and 11 were associated with VCs and PCs, respectively (**Figures 2D–F**). The cell identifies of ECs, VCs, and MCs were confirmed by RNA *in situ hybridization* (RIH) experiments using *G. pentaphyllum* leaves (**Figure 3**).

We observed significant cellular heterogeneity within the *G. pentaphyllum* shoot apexes and leaves, particularly for MCs (**Figures 2A–D**). For instance, MCs were segregated into 8 distinct clusters in leaves, exhibiting the highest heterogeneity among all the populations (**Figure 2A**), a phenomenon also reported in *Catharanthus roseus* leaves (Sun et al., 2023). Moreover, Gene Ontology (GO) enrichment analysis indicated substantial transcriptomic heterogeneity in the same cell type between shoot apexes and leaves (**Supplementary Figure 1**). Specifically, MCs from shoot apexes exhibited enrichment in processes associated with “ribosomal ribosome” and “mRNA binding”, reflecting their involvement in cell proliferation and growth during the rapid development of shoot apexes (**Supplementary Figure 1A**). In contrast, signature genes in MCs from leaves were enriched for processes related to “photosynthesis” and “chloroplast thylakoid membrane”, underscoring their role in photosynthesis (**Supplementary Figure 1B**). Additionally, shoot apex ECs displayed enrichment for processes related to “lipid catabolic process” and “lipid transport”, indicating robust lipid metabolism and transport in the shoot apexes (**Supplementary Figure 1C**). On the other hand, the enriched gene signals in ECs from leaves were associated with “response to stimulus” and “response to stress”, aligning with the role of leaves as sensory organs (**Supplementary Figure 1D**).

### 3.2 Spatiotemporal distribution of gypenoside biosynthesis in *G. pentaphyllum* shoot apexes and leaves

**Figure 4:**
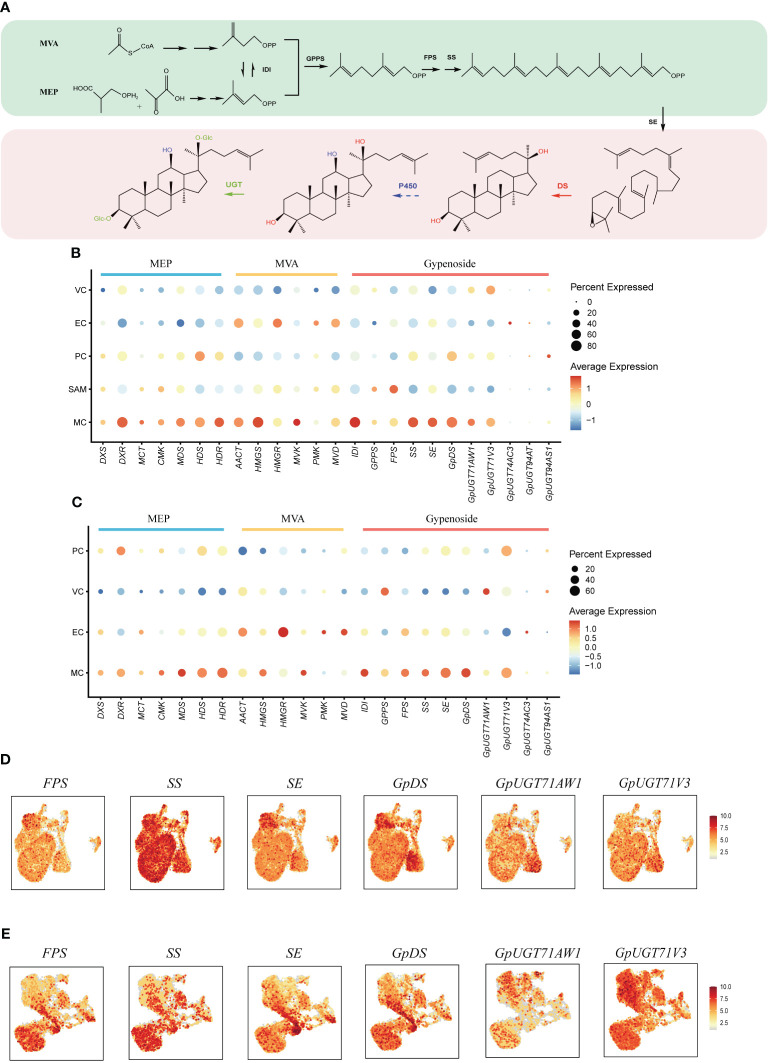
Spatial distribution of gypenoside biosynthesis in *G. pentaphyllum* shoot apexes and leaves. **(A)** The proposed gypenoside biosynthetic pathway. The full names of the selected genes are provided in **Supplementary Table 4**. **(B, C)** Cell type specificity of gypenoside biosynthesis in shoot apexes and leaves, respectively. **(D, E)** UMAP visualization of gene expression patterns involved in gypenoside biosynthesis in shoot apexes and leaves, respectively. Color intensity indicates the relative transcript level for the indicated gene in each cell.

Gypenosides originate from C5 isopentenyl building blocks produced through two primary pathways: the mevalonic acid pathway (MVA) and the methylerythritol phosphate pathway (MEP) (**Figure 4A**) (Vranova et al., 2013). Within both shoot apexes and leaves of *G. pentaphyllum*, MEP pathway genes demonstrated elevated expression in MCs compared to other cell types, while MVA pathway genes exhibited higher expression levels in both MCs and ECs (**Figures 4B, C**). Notably, MEP pathway genes exhibited varying expression levels in subtypes of MCs, with the highest expression observed in Cluster 7 of MCs in leaves. In contrast, this pattern was not observed in the shoot apexes (**Supplementary Figure 2**). This observation suggested that distinct MC subtypes possess unique capabilities for C5 isopentenyl unit biosynthesis. Furthermore, a tissue-level differential expression analysis of MEP and MVA pathway genes in shoot apexes and leaves was conducted. With the exception of the deoxyxylulose 5-phosphate synthase (DXS) and 4-hydroxy-3-methylbut-2-enyl diphosphate reductase (HDR) genes of the MEP pathway, the expression levels of the other genes in the shoot apex were greater than those in the leaves (**Supplementary Figure 3**). These findings suggest that C5 isopentenyl unit biosynthesis is potentially more active in the shoot apexes than in leaves of *G. pentaphyllum*.

The expression of the genes encoding isopentenyl diphosphate isomerase (IDI), which catalyzes the interconversion of the C5 units between isopentenyl pyrophosphate (IPP) and dimethylallyl pyrophosphate (DMAPP), was also elevated in MCs (**Figures 4B, C**). IPP and DMAPP are sequentially converted to dammarenediol-II through reactions mediated by geranyl pyrophosphate synthase (GPPS), farnesyl pyrophosphate synthase (FPS), squalene synthase (SS), squalene epoxidase (SE), and dammarenediol-II synthase (GpDS) (**Figure 4A**). *SS*, *SE* and *GpDS* exhibited preferential expression in MCs of both shoot apexes and leaves (**Figures 4D, E**). Both *GPPS* and *FPS* showed elevated transcription in MCs and SAMs of shoot apexes, whereas in leaves, *GPPS* expression was elevated in MCs and VCs, while *FPS* transcripts were enriched in MCs and ECs (**Figures 4B, C**). The dammarenediol-II skeleton underwent subsequent modifications to yield various gypenosides, a process mediated by cytochrome P450s (CYP450s) and UDP-glucosyltransferases (UGTs) (**Figure 4A**). While no CYP450s involved in gypenoside biosynthesis have been identified, five UGTs have been characterized thus far. *UGT71AW1*and *UGT71V3* demonstrated relatively greater expression in MCs in both shoot apexes and leaves, while other *UGT* transcripts were enriched in distinct cell types (**Figures 4B, C**). In summary, the majority of genes involved in gypenoside biosynthesis exhibited increased expression in the MCs of both shoot apexes and leaves, suggesting that MCs serve as the principal cellular sites for gypenoside biosynthesis.

### 3.3 Cell type-specific expression of TEs in shoot apexes and leaves

**Figure 5:**
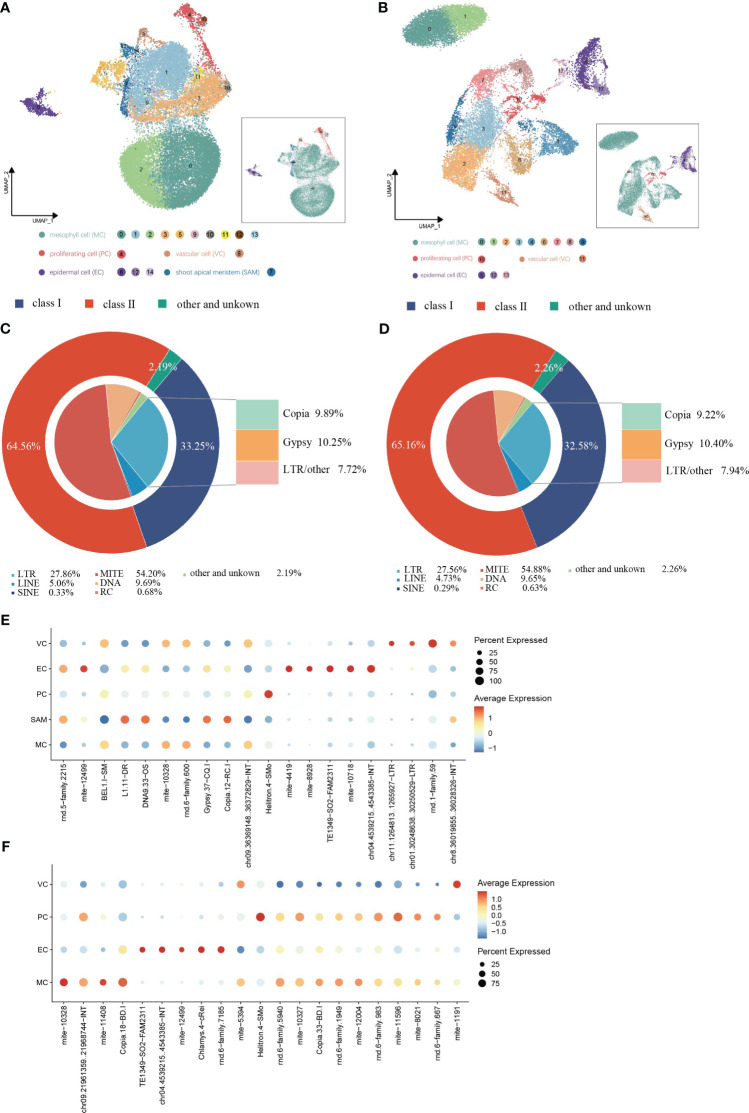
Spatial distribution of TE activity in (*G*) *pentaphyllum* shoot apexes and leaves. **(A, B)** UMAP visualization of cell clusters generated by the integrated gene/TE expression datasets from shoot apexes and leaves, respectively. Each dot represents an individual cell; colors indicate cell clusters. **(C, D)** Classification of the expressed TEs in shoot apexes and leaves, respectively. **(E, F)** Cell type-specific TE expression patterns in shoot apexes and leaves, respectively.

TE expression in single cells from shoot apexes and leaves was quantified using the scTE pipeline, generating gene/TE expression matrices for subsequent analysis. The resulting cell clusters in the shoot apexes and leaves were classified into four and five distinct cell types, respectively, consistent with the cell type outputs based solely on gene expression (**Figures 5A, B**; **Supplementary Figure 4**). The Sankey diagram illustrates that the identities of cells within each cell type were not significantly altered regarding both gene and TE expression (**Supplementary Figure 5**). In total, 17,376 expressed TE families were detected in shoot apexes, including 5,778 Class I TEs and 11,218 Class II TEs, while 15,404 TE expressed families were identified in leaves, including 5,019 Class I TEs and 10,037 Class II TEs (**Supplementary Tables 5, 6**). Among the TEs expressed in the shoot apexes and leaves, the LTR subclass comprised the highest proportion (28%) of Class I TEs, while the MITE subclass accounted for the highest proportion of Class II TEs, 54% in the shoot apexes and 55% in the leaves, respectively. (**Figures 5C, D**). These results suggested that TE expression profiles at the class and subclass levels are roughly comparable between shoot apexes and leaves.

In the leaves, 21 TE subfamilies exhibited cell type-specific expression, whereas in the shoot apexes, 20 TE subfamilies exhibited cell type-specific expression (**Figures 5E, F**; **Supplementary Tables 7, 8**). Notably, we found that most of cell type-specific TE subfamilies differed even within the same cell type between shoot apexes and leaves. However, five cell type-specific TE subfamilies (mite-10328, TE1349-SO2-FAM2311, chr04.4539215.4543385-INT, mite-12499, and Helitron.4-SMo) were present in both tissues, among which four TE subfamilies were specifically expressed in the same cell type across both leaves and shoot apexes. Interestingly, mite-12499 was specifically expressed in ECs and VCs in leaves, while in shoot apexes it was specifically expressed in ECs and SAMs (**Figures 5E, F**; **Supplementary Tables 7, 8**). These findings provide novel evidence for the spatiotemporal heterogeneity of TE expression within plants.

### 3.4 Stage-specific expression of TEs and genes involved in gypenoside biosynthesis during developmental trajectories

To establish coherent developmental trajectories, we initially integrated gene/TE expression datasets from shoot apexes and leaves, and the resultant dataset was used for cell clustering and cell identity annotation. Based on the distinctive expression patterns of cell-type markers, Clusters 10 and 11 were annotated as PCs, Cluster 14 as SAMs, cluster 15 as VCs, and Clusters 7,12, and 13 as ECs. Moreover, Clusters 0-6, 8, 9 and 16 were designated as MCs (**Supplementary Figure 6**).

**Figure 6:**
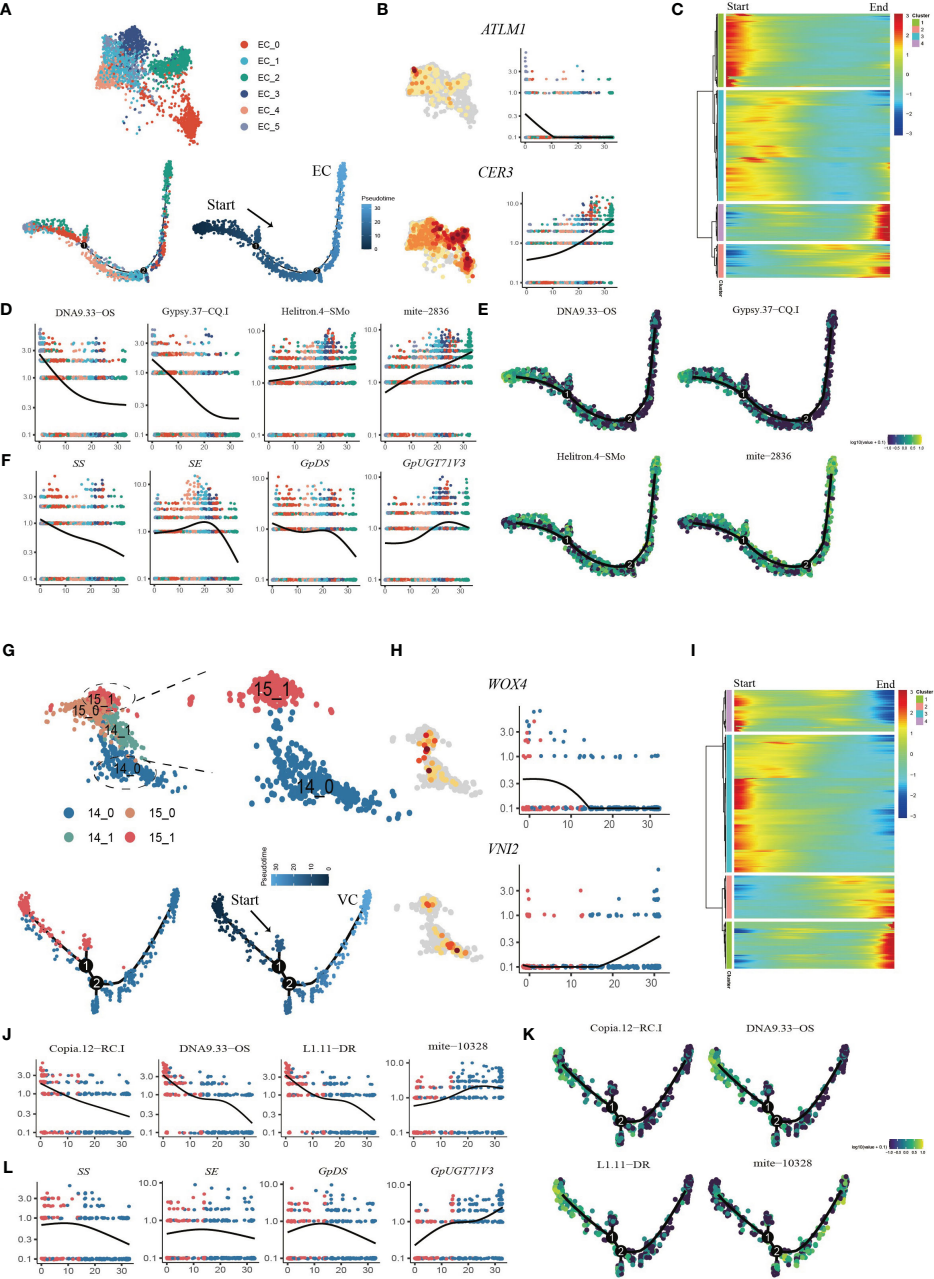
Reconstruction of the developmental trajectories of ECs and VCs. **(A)** UMAP visualization and cells distribution of ECs along the pseudotime trajectory. Each dot indicates a single cell, and the color of the cell indicates cell identities (below left). The color on the dot indicates the pseudotime score (below right). **(B)** UMAP plots of transcript accumulation and gene expression kinetics during EC pseudotime progression for marker genes, the full names of the selected genes are provided in **Supplementary Table 11**. **(C)** Heatmap showing the expression of pseudotime-dependent genes over the pseudotime trajectory of EC subclusters. **(D)** TE expression kinetics during EC pseudotime progression for representative TEs. **(E)** xpression patterns of representative TE are shown over the course of EC pseudo-time. **(F)** Gene expression kinetics during EC pseudotime progression for representative genes. **(G)** UMAP visualization and cells distribution of SAMs and VCs along the pseudotime trajectory. Each dot indicates a single cell, and the color of the cell indicates cell identities (below left). The color on the dot indicates the pseudotime score (below right). **(H)** UMAP plots of transcript accumulation and gene expression kinetics during VC pseudotime progression for marker genes, the full names of the selected genes are provided in **Supplementary Table 11**. **(I)** Heatmap showing the expression of pseudotime-dependent genes over the pseudotime trajectory of VC subclusters. **(J)** TE expression kinetics during VC pseudotime progression for representative TEs. **(K)** Expression patterns of representative TE are shown over the course of VC pseudotime. **(L)** Gene expression kinetics during VC pseudotime progression for representative genes.

Pseudotime trajectory analysis was applied to the EC populations (**Figure 6A**). We used the transcription factor ARABIDOPSIS THALIANA MERISTEM LAYER 1 (*ATML1*), which acts as a positive regulator of gibberellin (GA)-regulated epidermal gene expression, and *CER1*, which participates in the regulation of cuticle biosynthesis and wax accumulation, to distinguish between young and mature ECs (Bernard et al., 2012; Zuch et al., 2022). The developmental trajectory was initiated from Cluster EC_5, followed by EC_0, EC_4, EC_1, and EC_3, with EC_2 cells positioned at the terminus of the trajectory, according to the expression patterns of *AMTL1* and *CER3* along the developmental trajectory (**Figure 6B**; **Supplementary Figures 7A, B**). GO term analysis revealed that during the initial stages of the developmental trajectory (modules 1 and 3), there was an overrepresentation of genes associated with the regulation of meristem growth and cell growth. This observation aligned with the expected biological functions attributed to early ECs during development. Conversely, at the terminus of the trajectory, module 4 encapsulated the expression of genes associated with the abiotic stress response, aligning with the heightened sensitivity of mature epidermal cells to environmental stimuli (**Figure 6C**; **Supplementary Table 9**). The dynamic expression patterns of TEs with specifically high expression in ECs were examined throughout the course of EC development (**Supplementary Figure 8**). Specifically, DNA9.33.OS, Gypsy.37-CQ.1 and L1.11.DR exhibited downregulated expression in the late stages of EC development, while Chlamys.4-cRei, mite-2836 and Helitron.4-SMo exhibited upregulated expression in the late stages of EC development (**Figures 6D, E**; **Supplementary Figure 8**). These findings suggest that these TEs may regulate epidermal cell fate specification. We also analyzed the dynamic expression patterns of genes involved in the gypenoside biosynthetic pathway during epidermal development (**Supplementary Figure 9**). Intriguingly, the key genes (*FPS*, *SS*, *SE*, and *GpDS*) responsible for synthesizing the triterpene skeleton dammarenediol-II were expressed at higher levels in younger ECs, and progressively diminished along the developmental trajectory. Conversely, the expression of *GpUGT71V3* exhibited slight upregulation in the later stages of EC development (**Figure 6F**). Moreover, the expression of *GpUGT71AW1* exhibited slight upregulation in the early stages of EC development and then decreased in the middle stages of EC development but exhibited upregulation in the late stages of EC development.

Pseudotime analysis was also applied to the VC and SAM populations to determine the cell differentiation states during VC development (**Figure 6G**; **Supplementary Figure 7C, D**). First, the VCs were reassigned into two subclusters, among which Cluster 14_0 mainly expressed orthologues of *Arabidopsis* xylem cell markers, namely, *WOX4*, *TDR*, *ATHB8* and *VINI2* (Hirakawa et al., 2010; Fukuda and Ohashi-Ito, 2019) (**Figure 6H**; **Supplementary Figure 10A**). The SAM population (Cluster15) was reassigned into two subclusters, and the 15_1 subcluster was highly expressed SAM marker genes (**Supplementary Figure 10B**). We extracted subcluster 14_0 with subcluster 15_1 to construct a putative developmental trajectory from the SAM to the xylem (**Figure 6G**). GO enrichment of DEGs on the developmental trajectory was performed. The enriched gene signal at the beginning of the pseudotime axis (modules 3 and 4) was consistent with the functions of cell proliferation and nucleic acid replication in primary growth (**Figure 6I**; **Supplementary Table 10**). At the end of the trajectory, module 1 captured the expression of genes involved in material transport, the abiotic stress response and lignin catabolism, consistent with the function of mature xylem (**Figure 6I**; **Supplementary Table 10**). Interestingly, we found that the TE subfamilies Copia.12-RC.I DNA9.33-OS and L1.11-DR were highly expressed in the early stages of cell development, while seven TE subfamilies from mite subclass were high expressed in the later stage of development (**Figures 6J, K**; **Supplementary Figure 11**). In addition, we also examined the variation in the expression of genes involved in the biosynthesis of gypenosides (**Supplementary Figure 12**). The key genes *SS*, *SE* and *DS* involved in the synthesis of the triterpenoid skeleton were highly expressed in the early stage of development and then sharply decreased (**Figure 6L**). Only *GpUGT71V3* and *GPPS* were upregulated along the trajectory and were highly expressed in mature xylem cells (**Supplementary Figure 12**). Taken together, the results of pseudotime analysis provided novel insights into the dynamic process of xylem development and variations in the expression of specific TEs and genes involved in gypenoside biosynthesis during the transition of cell states.

## 4 Discussion

Over the past five years, plant science has been revolutionized by enabling transcriptome profiling at single-cell resolution via scRNA-seq, particularly in the exploration of cellular heterogeneity and developmental trajectories (Bawa et al., 2023). However, its applications in studying plant specialized metabolism and TE activities remain limited. In this study, by constructing single-cell atlases of *G. pentaphyllum* shoot apexes and leaves, we revealed the spatiotemporal distribution of gypenoside biosynthesis. More importantly, we provide the first landscape of TE activities in plant tissues at single-cell resolution. We found an imbalanced distribution of TE activities among different cell types and during leaf development.

Cell clustering and annotation constitute foundational steps in the analysis of scRNA-seq data. However, accurately annotating all cell types in the investigated tissues remains challenging, particularly for nonmodel plants. In this study, the absence of trichome cells limits the ability to construct a more comprehensive transcriptomic profile. A relatively large cell size (approximately 150 μm) may lead to fragmentation of these cells, preventing them from passing through the cell strainer during protoplast preparation (**Figure 1E**). A similar mechanism has been proposed to explain the loss of laticifer cells during the protoplasting of C. roseus leaves (Sun et al., 2023). Despite the progress in plant science, the availability of specialized annotation tools and reference datasets for plants lags behind that of animals. Currently, multiple software tools exist for automated cell type annotation of scRNA-seq data, such as SingleR (Aran et al., 2019), scVI (Lopez et al., 2018), scDHA (Tran et al., 2021), and CIForm (Xu et al., 2023a). These tools primarily rely on reference data for animals like humans and mice, leaving a gap in annotation resources for plants. As plant science progresses, it is anticipated that more specialized annotation tools and reference datasets for plants will emerge, catering to the needs of plant research. In addition, the effective isolation of plant protoplasts is often influenced by various factors, including cell size, cell shape, the relative position of cells within tissues (surface or inner layer), the biochemical composition of the cell wall, and the developmental stage of the cells are situated (Shaw et al., 2021). Single-nucleus RNA-seq may provide an alternative approach to capture additional cell types through nucleus isolation rather than protoplasting. Nucleus preparation is simpler and less impacted by cellular characteristics or positional context within tissues. The lack of suitable cell marker genes remains a significant challenge in cell annotation for nonmodel plants. Spatial transcriptomics can provide additional insights into cell type annotation due to its ability to capture spatial information (Tian et al., 2023). Moreover, with the assistance of spatial information, subtypes such as palisade versus sponge mesophyll cells or upper versus lower epidermal cells could be resolved (Xia et al., 2022).With the rapid advancement of single-cell transcriptomics technology, emerging tools have opened new avenues for diversified data analysis and utilization. For instance, STGRNS effectively infers cell-specific gene regulatory networks through the analysis of single-cell transcriptomic data (Xu et al., 2023b), while scmFormer delves into the intricate relationships between gene expression and protein levels by integrating single-cell proteomic data (Xu et al., 2024). Looking ahead, the integration of single-cell transcriptomics with single-cell multi-omics technology will become a trend, providing us with more comprehensive and profound cellular analysis tools.

Compartmentalization of specialized metabolism is a widespread phenomenon across the plant kingdom that occurs at multiple hierarchical levels, ranging from the molecular to the organ level (Watkins and Facchini, 2022). This organizational strategy provides distinct advantages to plants, including the enhancement of metabolic output, alleviation of enzyme inhibition, and elimination of autotoxicity. Notably, among these levels of compartmentalization, the exploration of multicellular compartmentalization has been limited. ScRNA-seq has emerged as a potent tool for dissecting the multicellular compartmentalization of plant specialized metabolism. Utilizing this technology, it has been demonstrated that the vinblastine biosynthetic pathway in *C. roseus* leaves can be compartmentalized into distinct cell types, namely IPAP cells, ECs, and ICs (Li et al., 2023; Sun et al., 2023). However, we analyzed the expression patterns of the characterized genes involved in the gypenosides biosynthesis pathway at single-cell resolution to explore the spatial distribution of this pathway in *G. pentaphyllum* shoot apexes and leaves revealed that gypenoside biosynthesis is predominantly localized in MCs. Similarly, taxol biosynthesis was observed in MCs of *Taxus* leaves, as evidenced by the scRNA-seq data analysis (Zhan et al., 2023). These findings suggest that the biosynthesis of both gypenoside and taxol does not involve multicellular compartmentalization. Consequently, the extent to which specialized metabolic pathways are compartmentalized at the cellular level in the plant kingdom remains to be explored.

In this study, we conducted the first examination of single-cell TE expression in plants. TEs act as cis-regulatory elements and play pivotal roles in the regulation of gene expression (Marand et al., 2021). Furthermore, TE transcripts can also regulate host gene expression and reshape transcriptomes. Compared to those in animals, investigations into TE expression in plants have been scarce and confined to tissue-level analyses. Insertion time analysis unequivocally established the presence of a substantial population of young and transcriptionally active TEs within the *G. pentaphyllum* genome. Motivated by this discovery, we investigated TE expression at single-cell resolution in *G. pentaphyllum* shoot apexes and leaves. Our findings revealed that distinct TE families are expressed themselves in specific cell types, with certain TEs exhibiting dynamic expression patterns during the development of ECs and VCs. The precise mechanisms through which TE expression influences developmental processes warrant thorough investigation. Notably, we observed variation in the expression of genes involved in gypenoside biosynthesis during EC and VC development. However, whether TE expression participates in regulating pathway gene expression should be further explored. In conclusion, our study not only reveals the intricate landscape of TE expression at the single-cell level in plants but also opens avenues for investigating the roles of TE expression in regulating cell differentiation and development, as well as in specialized metabolism.

## 5 Conclusions

In this study, we generated single-cell transcriptome atlases of *G. pentaphyllum* leaf and shoot apexes. By analysis of the expression patterns of genes involved in gypenoside biosynthesis across distinct cell types, we observed that a majority of the genes associated with the gypenoside biosynthesis exhibited heightened expression in MCs. Additionally, we devised a novel single-cell transcriptome atlas of *G. pentaphyllum* by integrating gene and TEs expression data. Our analysis of TE expression profiling identified some TE families that are specific to cell types, tissues, or developmental stages, which indicated the potentially pivotal roles in cell differentiation and development. The scRNA-seq data presented in this study offers a valuable resource for investigating plant physiology and understanding the spatiotemporal distribution of specialized metabolism in this species.

## Data availability statement

The original contributions presented in the study are publicly available. This data can be found here: https://www.ncbi.nlm.nih.gov/search/all/?term=PRJNA1064230, https://www.ncbi.nlm.nih.gov/bioproject/PRJNA1030183.

## Author contributions

RL: Writing – original draft, Methodology, Validation. KD: Writing – original draft, Data curation, Formal Analysis, Visualization. CZ: Writing – original draft, Data curation, Formal Analysis, Visualization. XS: Writing – original draft. LY: Resources, Writing – original draft. SW: Resources, Writing – original draft. ZL: Investigation, Writing – original draft. ZS: Investigation, Writing – original draft. JW: Project administration, Writing – original draft. YL: Writing – original draft, Methodology, Software. BG: Project administration, Supervision, Writing – original draft. CS: Writing – original draft, Investigation, Project administration, Resources, Supervision.
