# Supplementary material for: Single-cell transcriptome profiling reveals the spatiotemporal distribution of triterpenoid saponin biosynthesis and transposable element activity in Gynostemma pentaphyllum shoot apexes and leaves
Source: Front Plant Sci. 2024 May 8;15:1394587. doi: 10.3389/fpls.2024.1394587 (PMC11109411; doi:10.3389/fpls.2024.1394587)
Supplement: Supplementary file 1 [file DataSheet_1.docx]

**Supplementary Figures 1-11.**


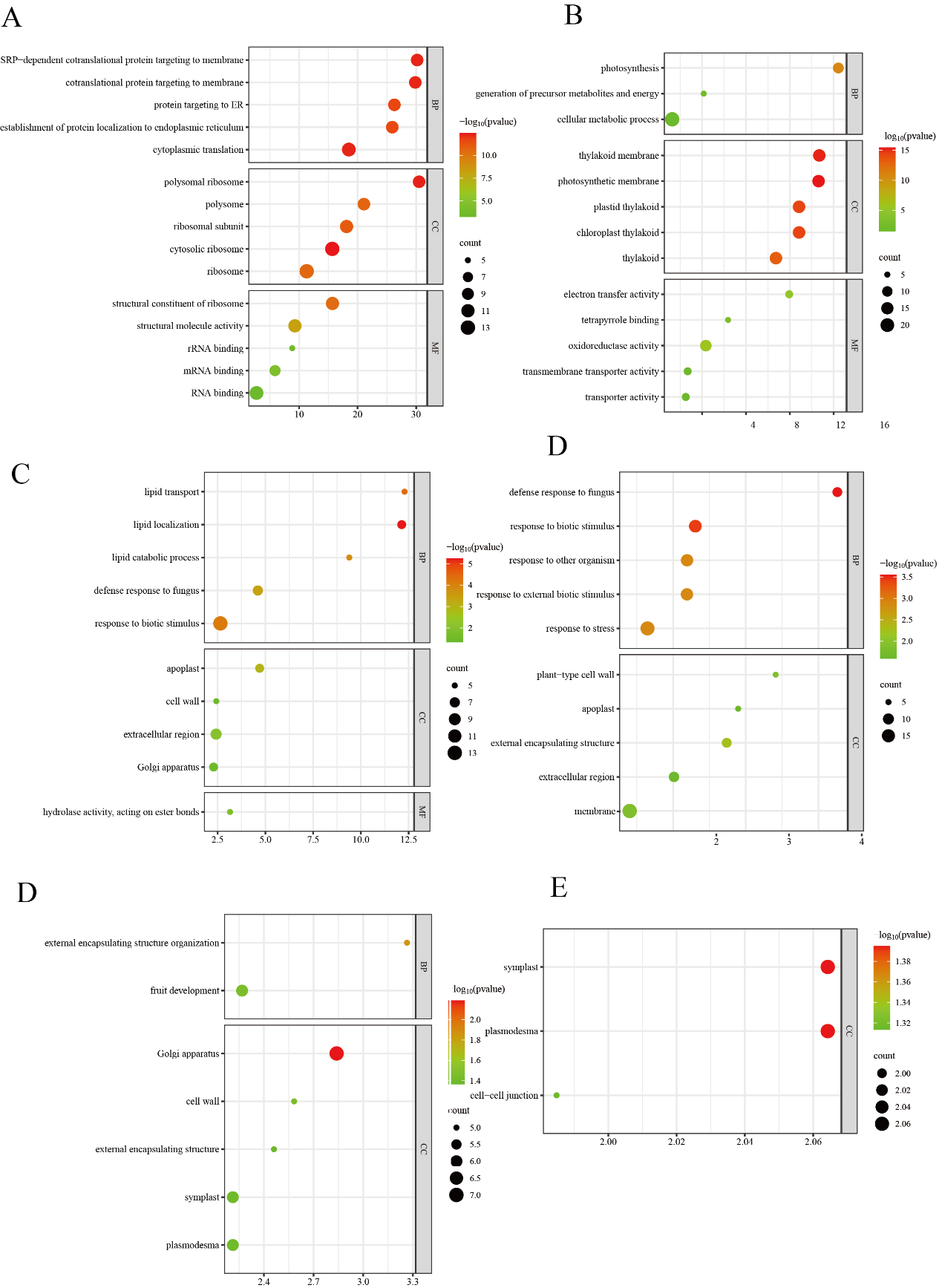


**Supplementary Figure 1. Heterogeneity of MCs, ECs, and VCs in shoot apexes (left) and leaves (right).**

**(A and B)** GO analysis of MCs; **(C and D)** GO analysis of ECs; **(E and F)** GO analysis of VCs. The top 30 Differentially Expressed Genes (DEGs), calculated by avg_logFC, were utilized for GO enrichment (Supplementary Tables 13 and 14), and the top five GO terms, based on -log_10_p-values, were chosen for visualization.


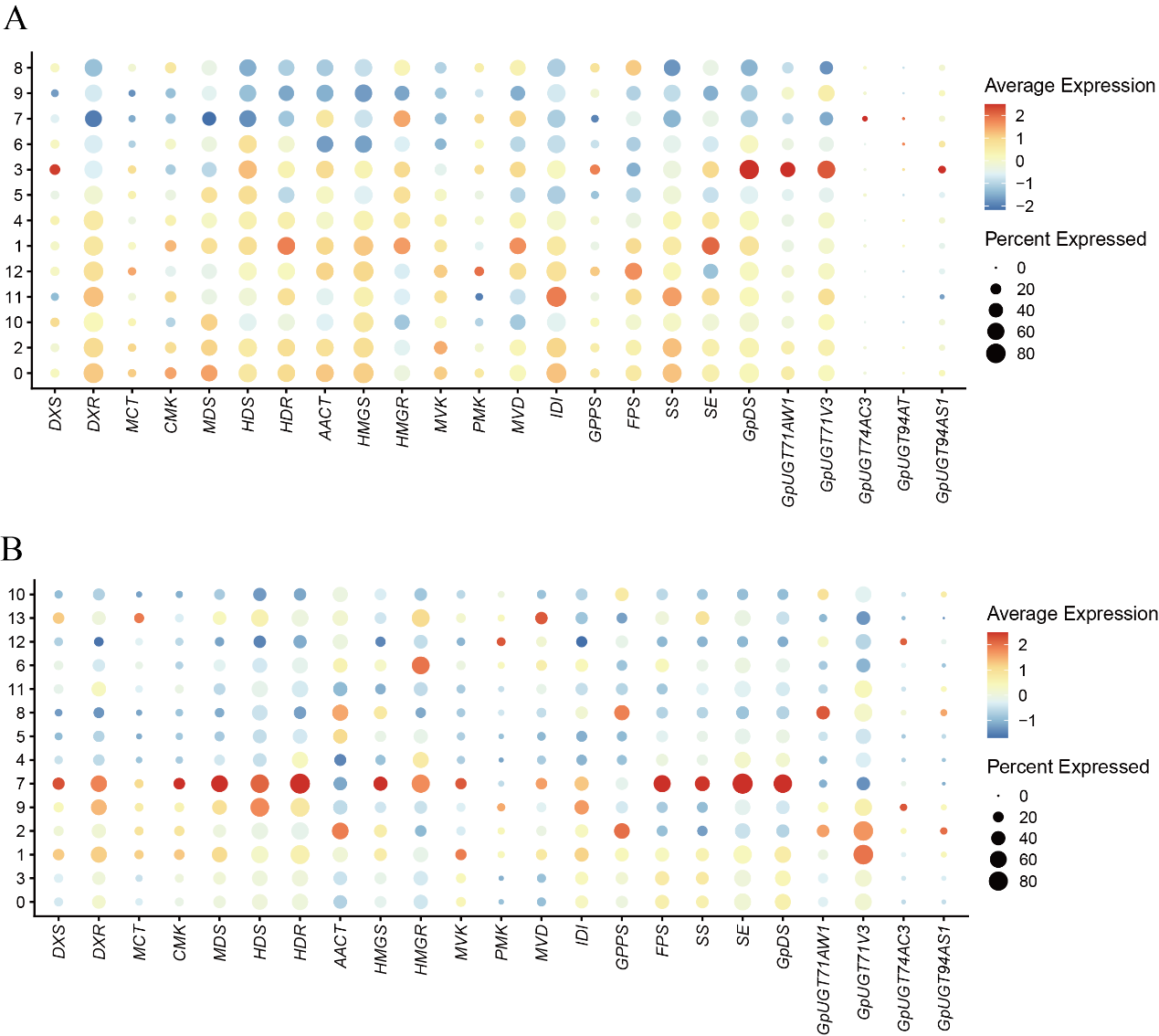


**Supplementary Figure 2. Cell cluster-specific expression of genes involved in the gypenoside biosynthetic pathway.**

**(A)***G. pentaphyllum* shoot apexes; **(B)** *G. pentaphyllum* leaves**.**


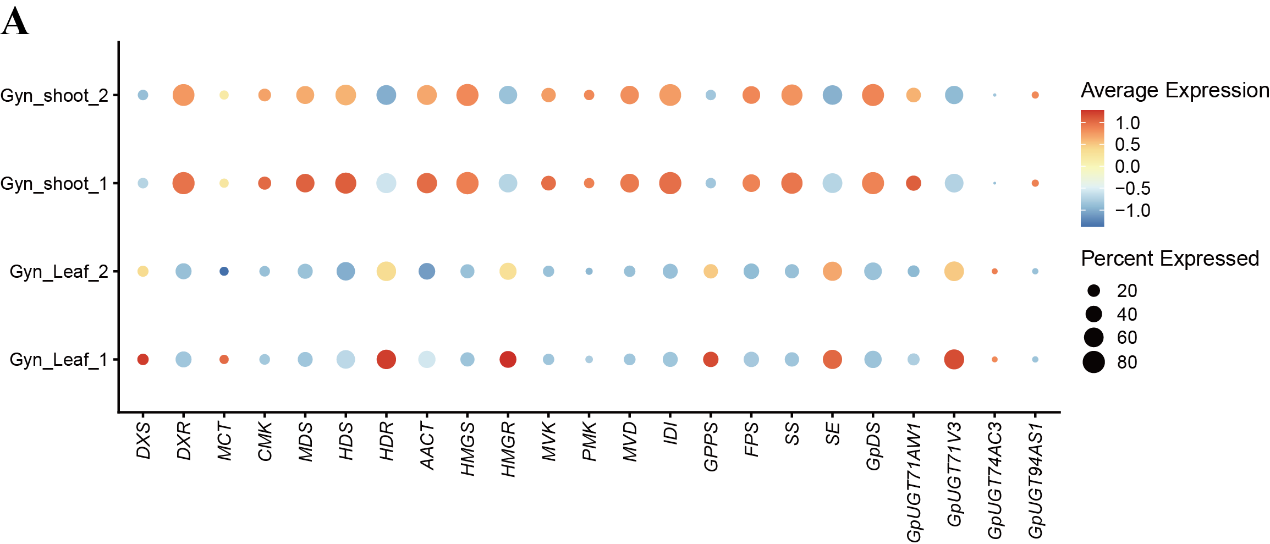


**Supplementary Figure 3. Comparison of the expression levels of genes involved in the gypenoside biosynthetic pathway between *G. pentaphyllum* shoot apexes and leaves.**


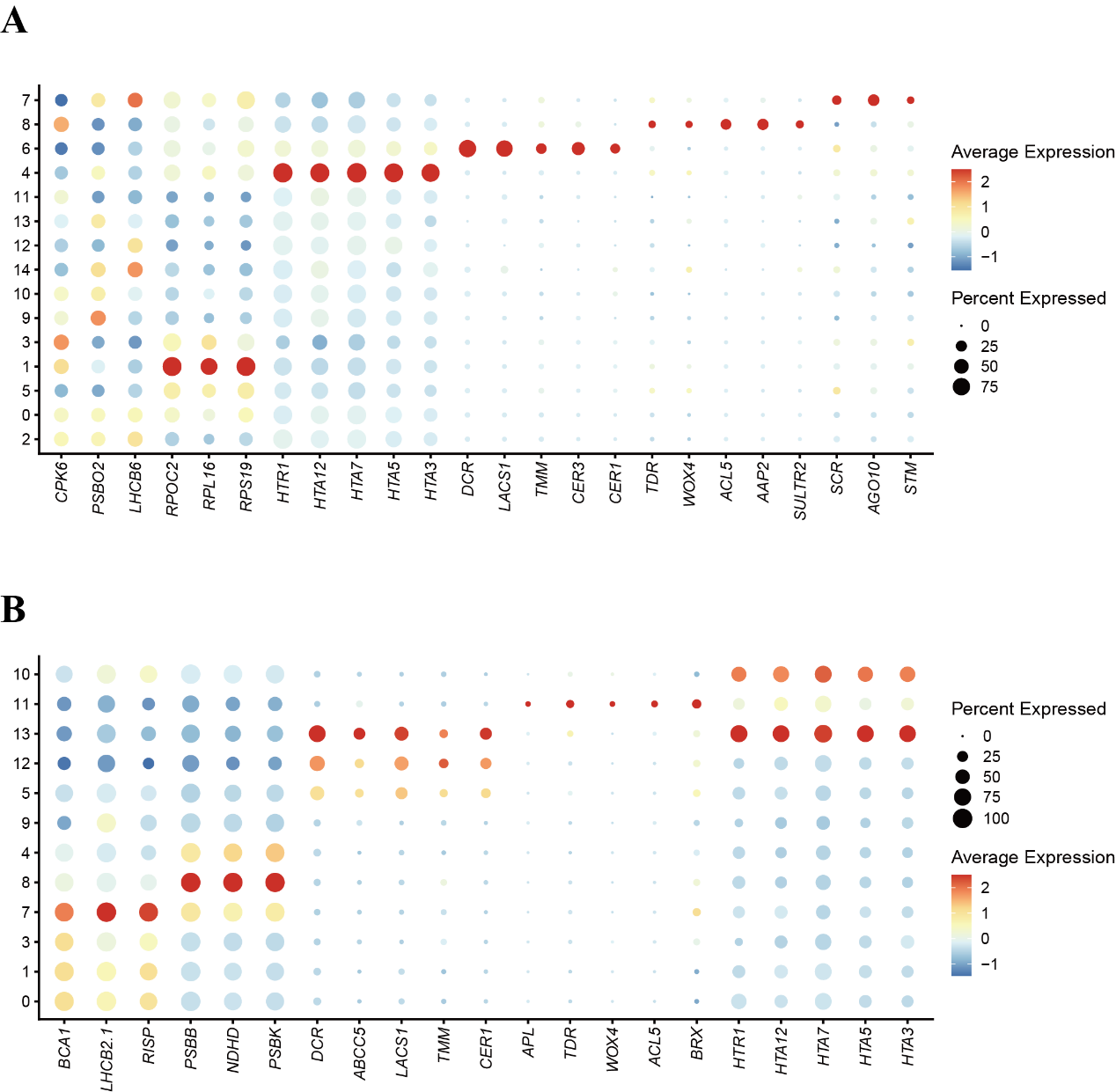


**Supplementary Figure 4. Expression patterns of representative cluster-specific marker genes.**

**(A)** *G. pentaphyllum* shoot apexes; **(B)** *G. pentaphyllum* leaves**.**

Dot diameter indicates proportion of cells in each cluster expressing each gene. The full names of the selected genes are given in Supplementary Table 3.


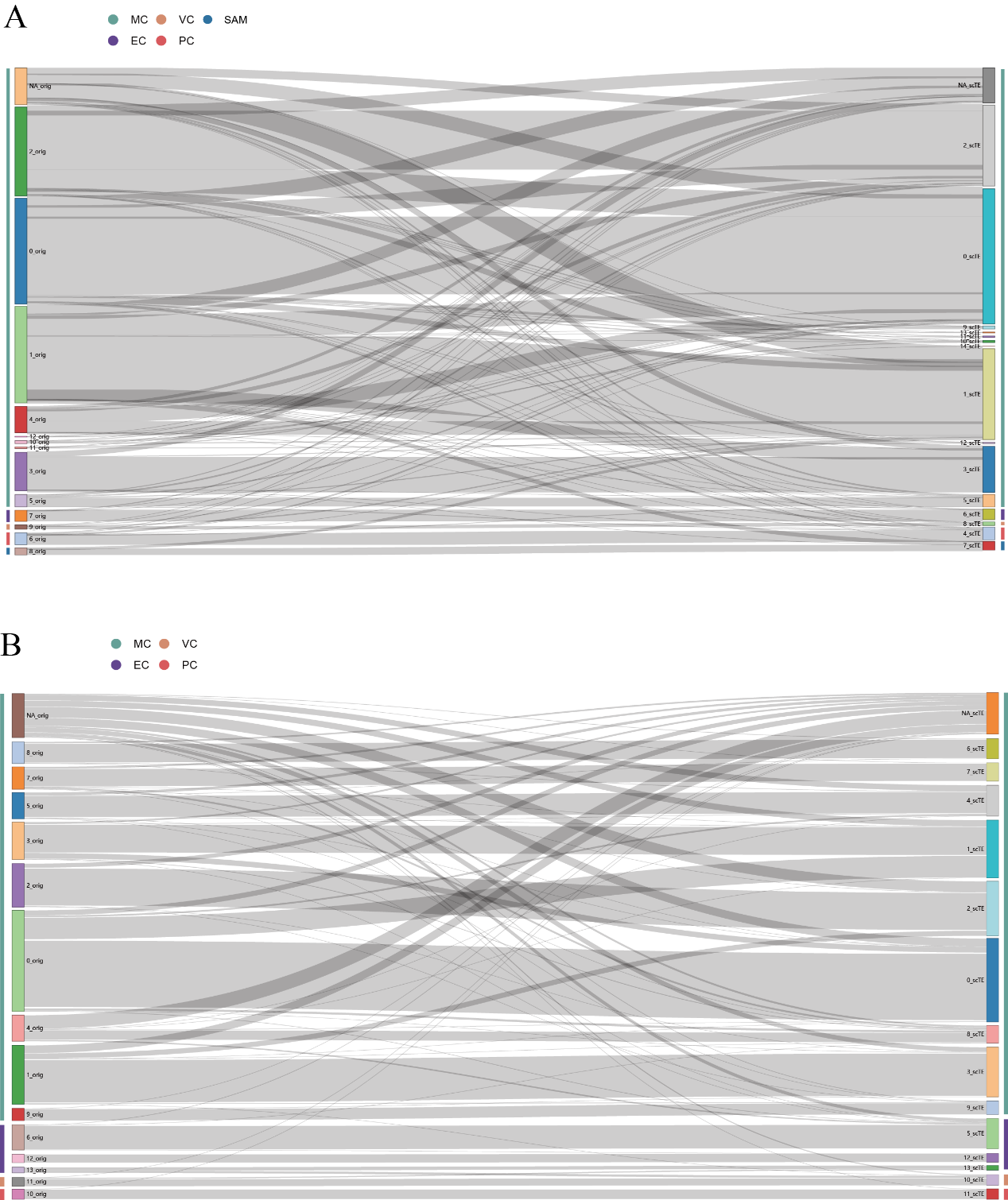


**Supplementary Figure 5. Comparison between clustering cells based on a gene expression matrix (left ) and a gene/TE expression matrix (right).**

**(A)** *G. pentaphyllum* shoot apexes; **(B)** *G. pentaphyllum* leaves


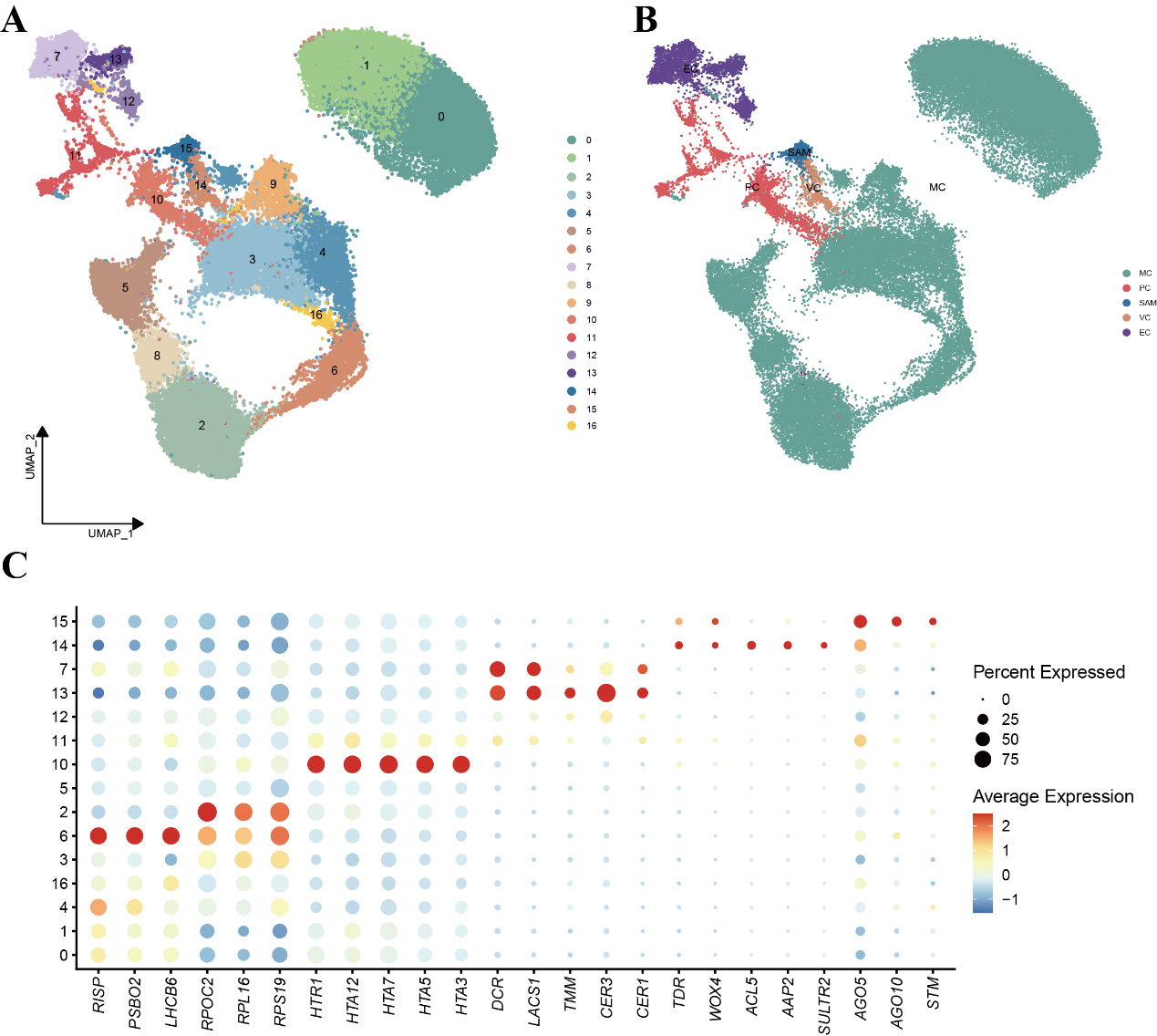


**Supplementary Figure 6. Identification of cell clusters from integrated gene/TE expression data of shoot apexes and leaves.**

**(A)** UMAP visualization of 17 cell clusters. Dots represent individual cells; color denotes cell cluster.

**(B)** UMAP visualization of five broad populations. Color represents population type.

**(C)** Expression patterns of representative cluster marker genes. Dot diameter indicates proportion of cells in each cluster expressing each gene. The full names of the selected genes are given in Supplementary Table 3.


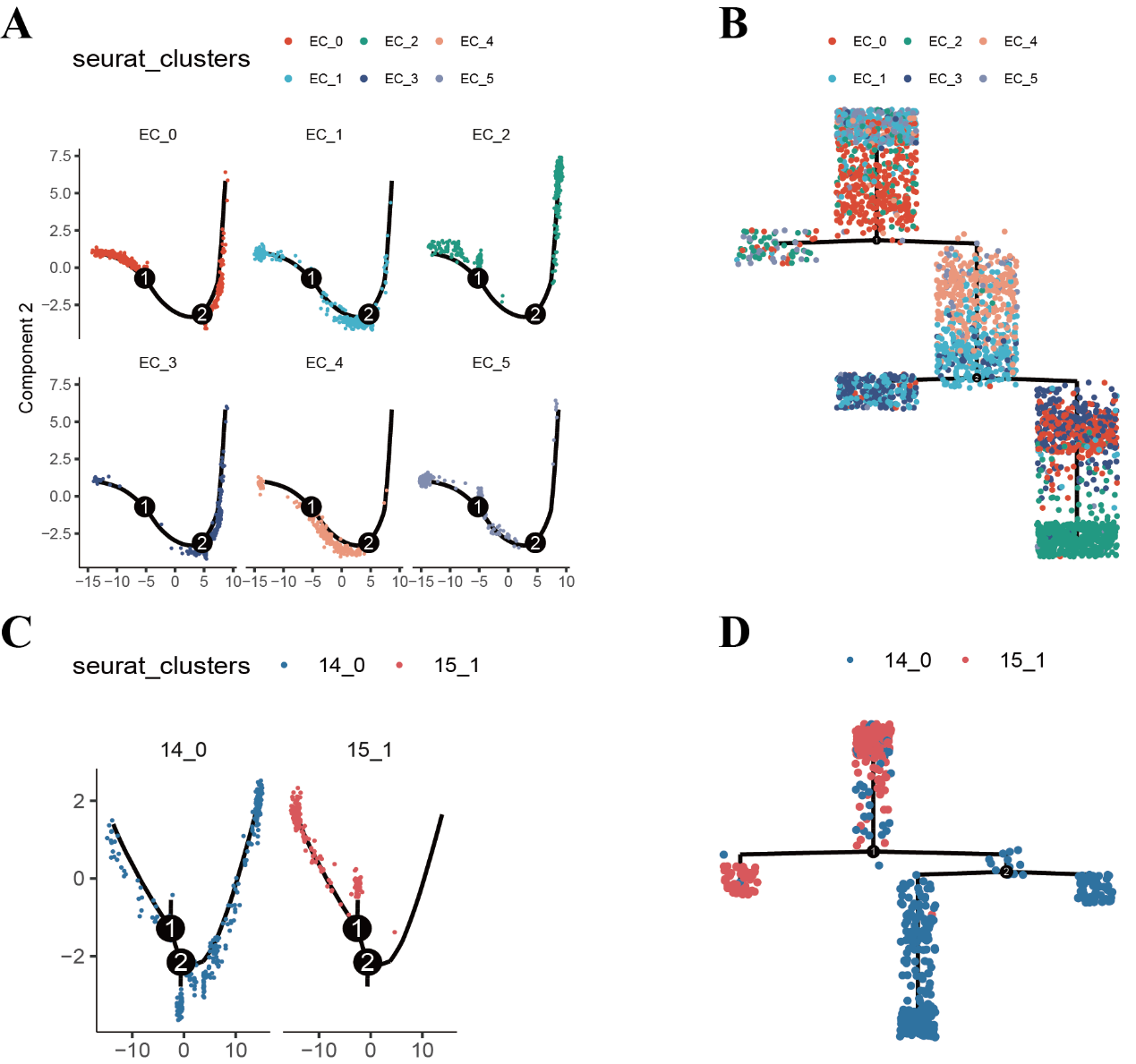


**Supplementary Figure 7. The developmental trajectories of ECs and VCs**.

**(A)** UMAP visualization of EC subcluster distribution along the pseudotime trajectory. Each dot represents a single cell.

**(B)** Tree diagram of the continuous EC differentiation trajectory over pseudotime. Branches indicate developmental stages; dots represent single cells.

**(C)** UMAP visualization of SAM and VC subcluster distribution along the pseudotime trajectory. Each dot represents a single cell.

**(D)** Tree diagram of the continuous SAM and VC differentiation trajectory over pseudotime. Branches indicate developmental stages; dots represent single cells.


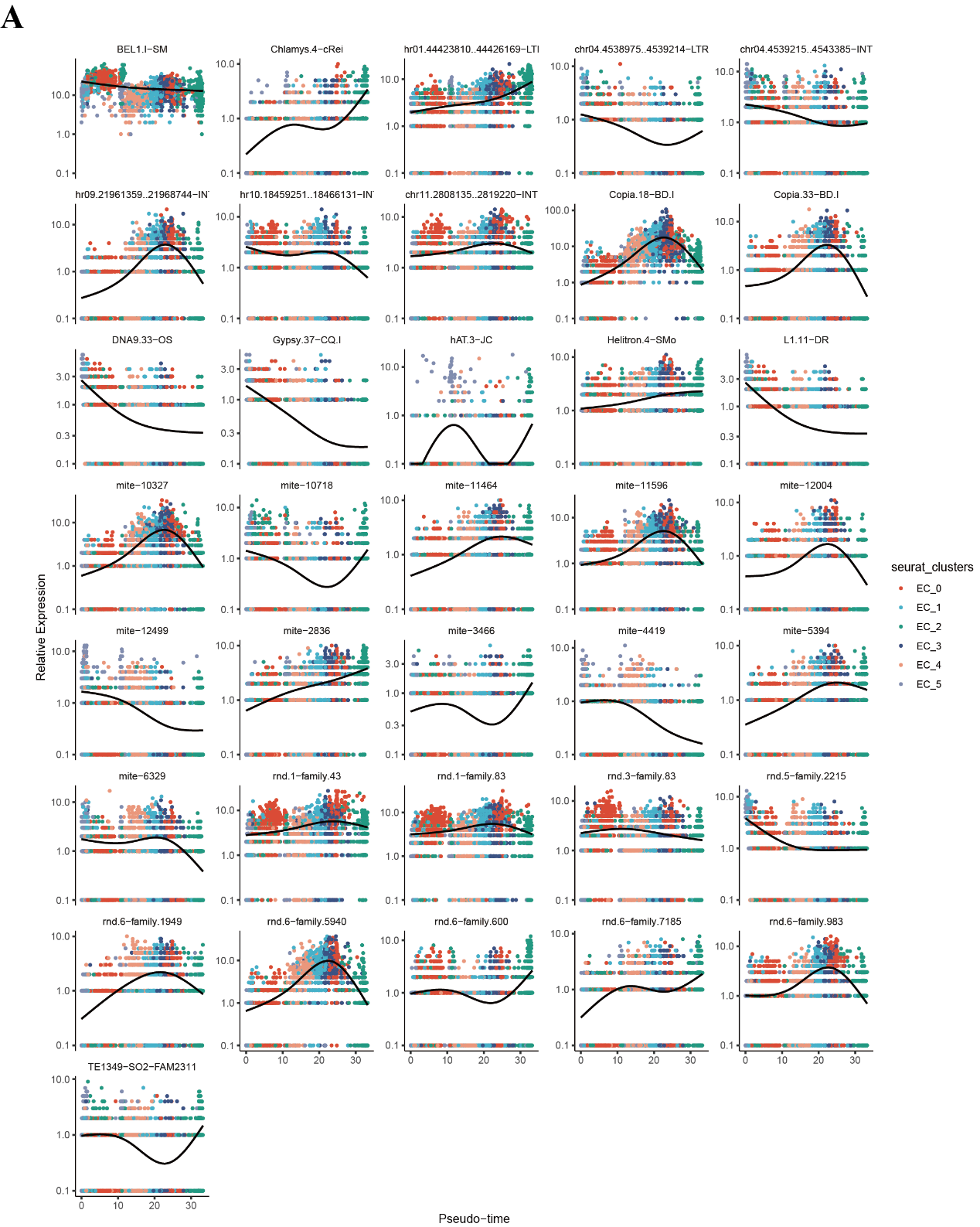


**Supplementary Figure 8. The dynamic expression patterns of the representative TEs during EC pseudotime progression.**


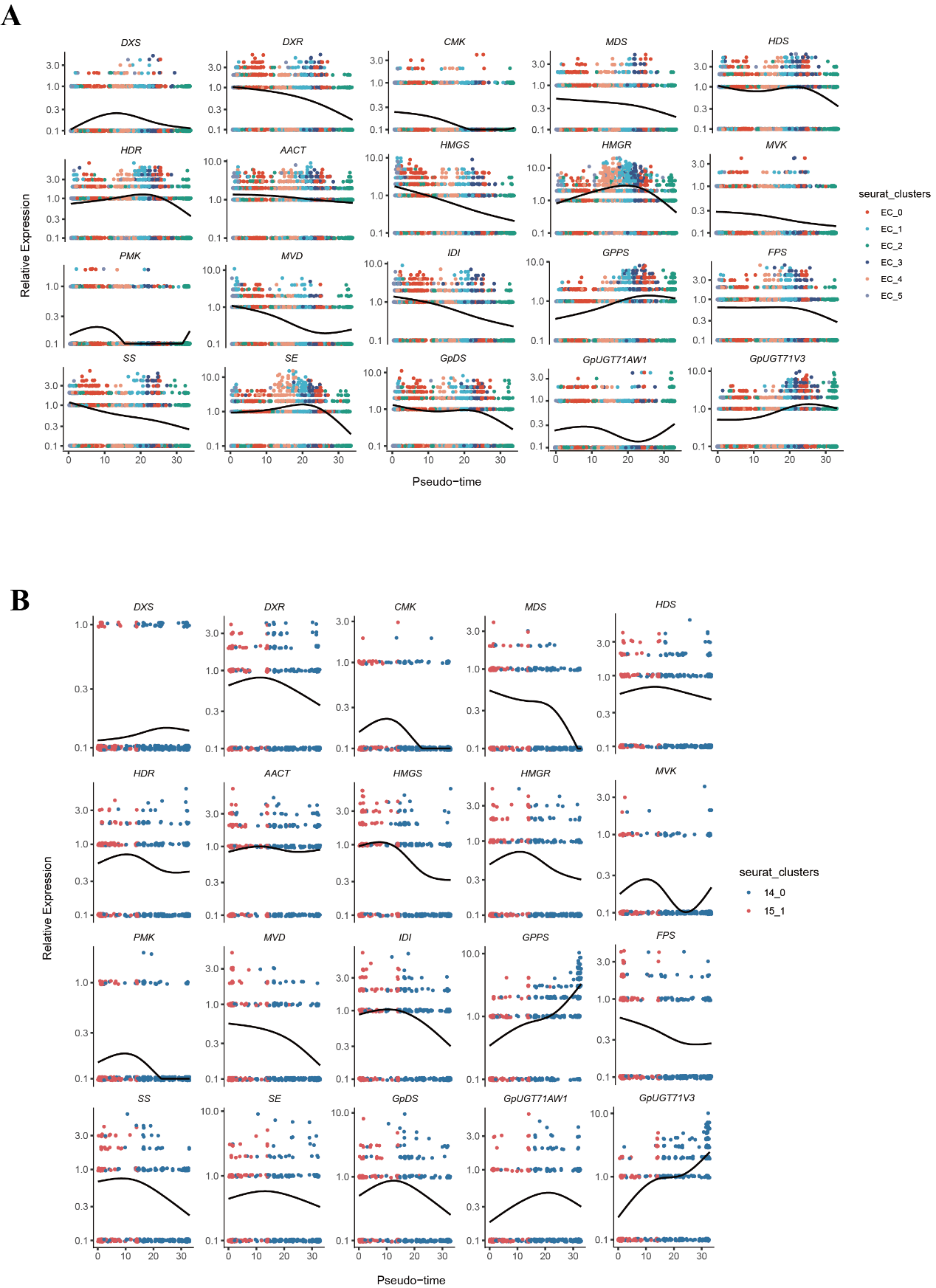


**Supplementary Figure 9. The dynamic expression patterns of genes involved in the gypenoside biosynthetic pathway during EC pseudotime progression.**


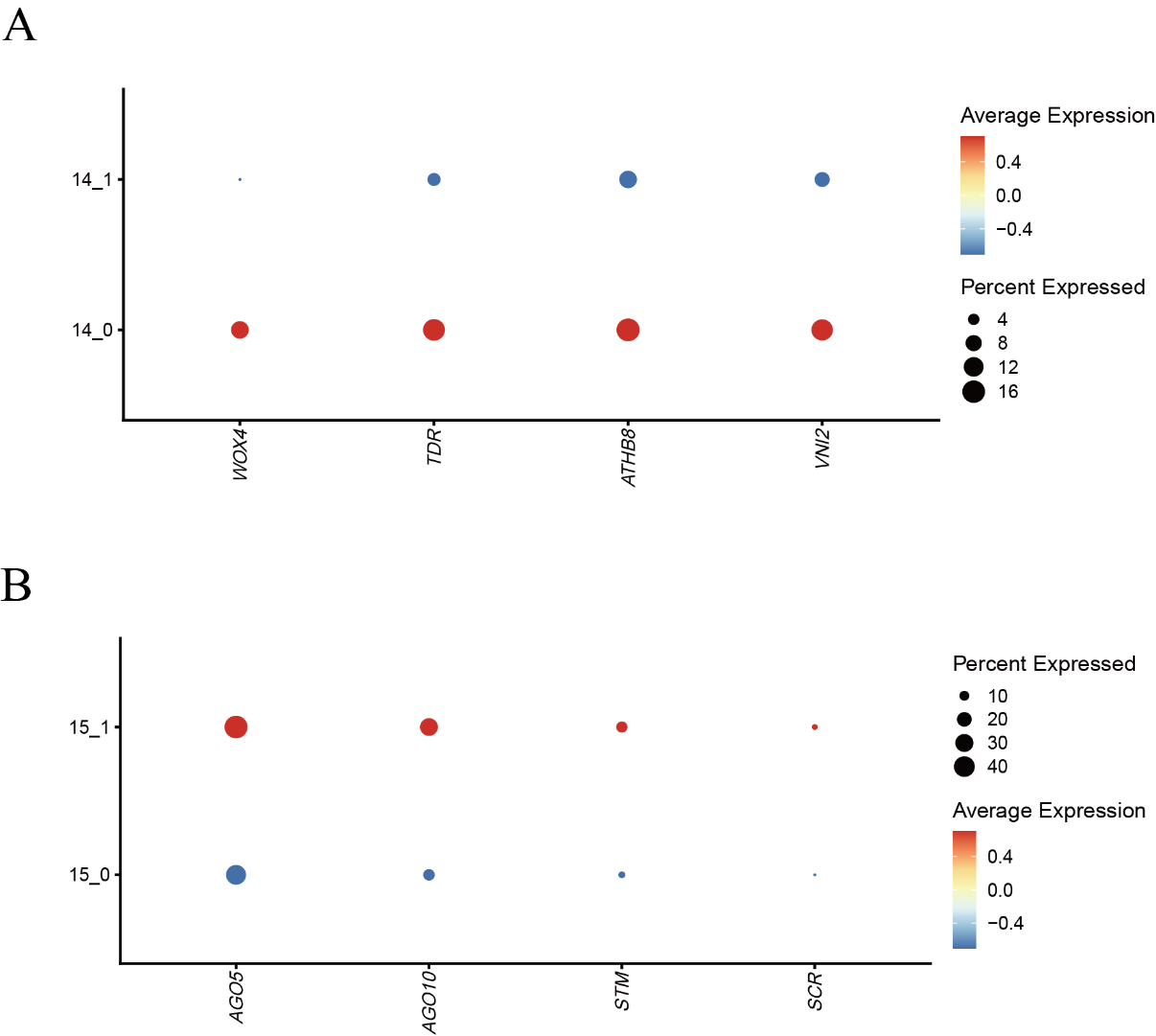


**Supplementary Figure 10. Expression patterns of cluster-specific marker genes.**

**(A)** Expression patterns of xylem cell markers in VC subcluster. The dot diameter represents the proportion of cluster cells expressing a given gene. The full names of selected genes are provided in Supplementary Table 15.

**(B)** Expression patterns of SAM markers in SAM subcluster. The dot diameter represents the proportion of cluster cells expressing a given gene. The full names of selected genes are provided in Supplementary Table 3.


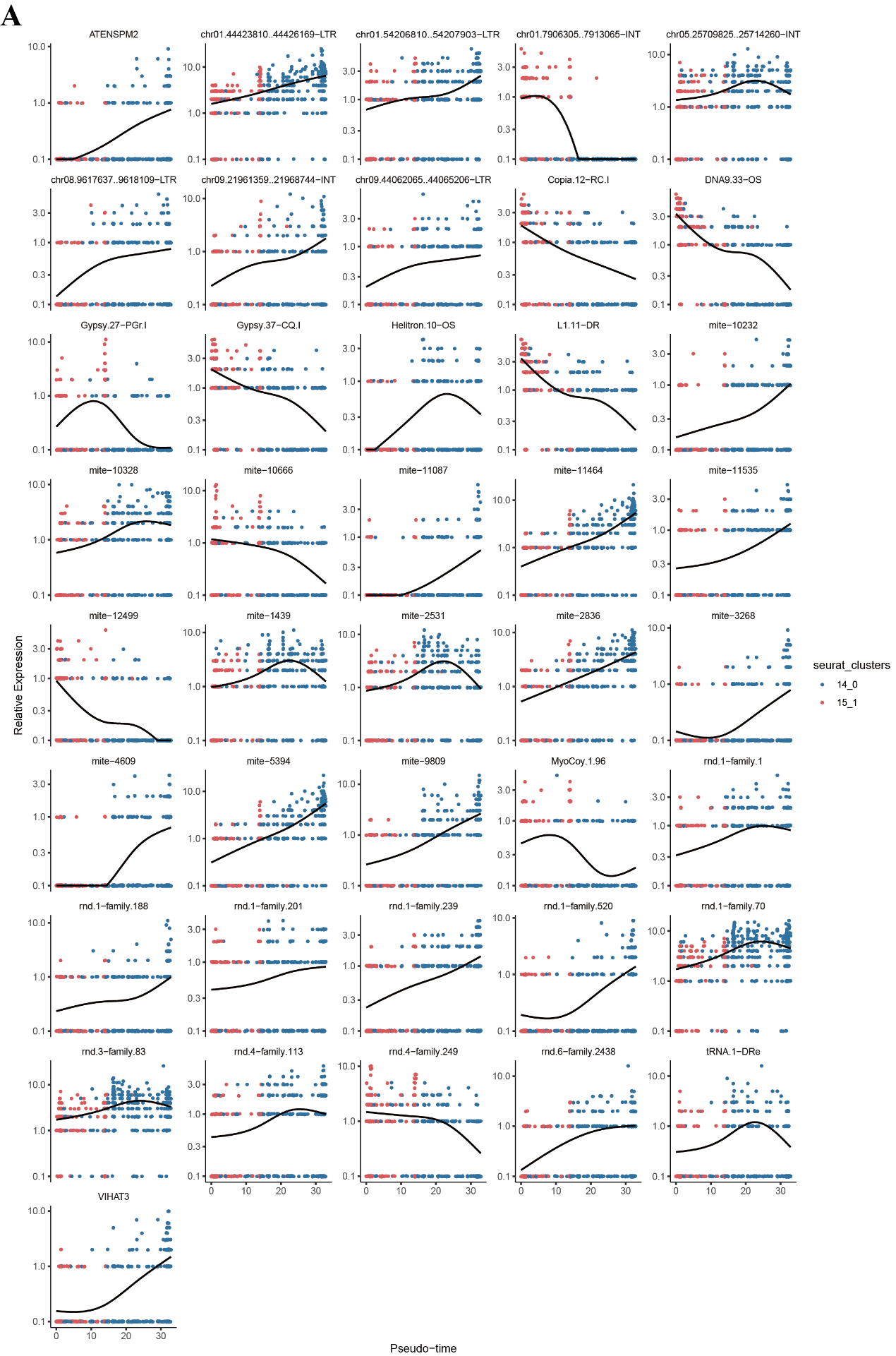


**Supplementary Figure 11. The dynamic expression patterns of the representative TEs during** **VC pseudotime progression.**


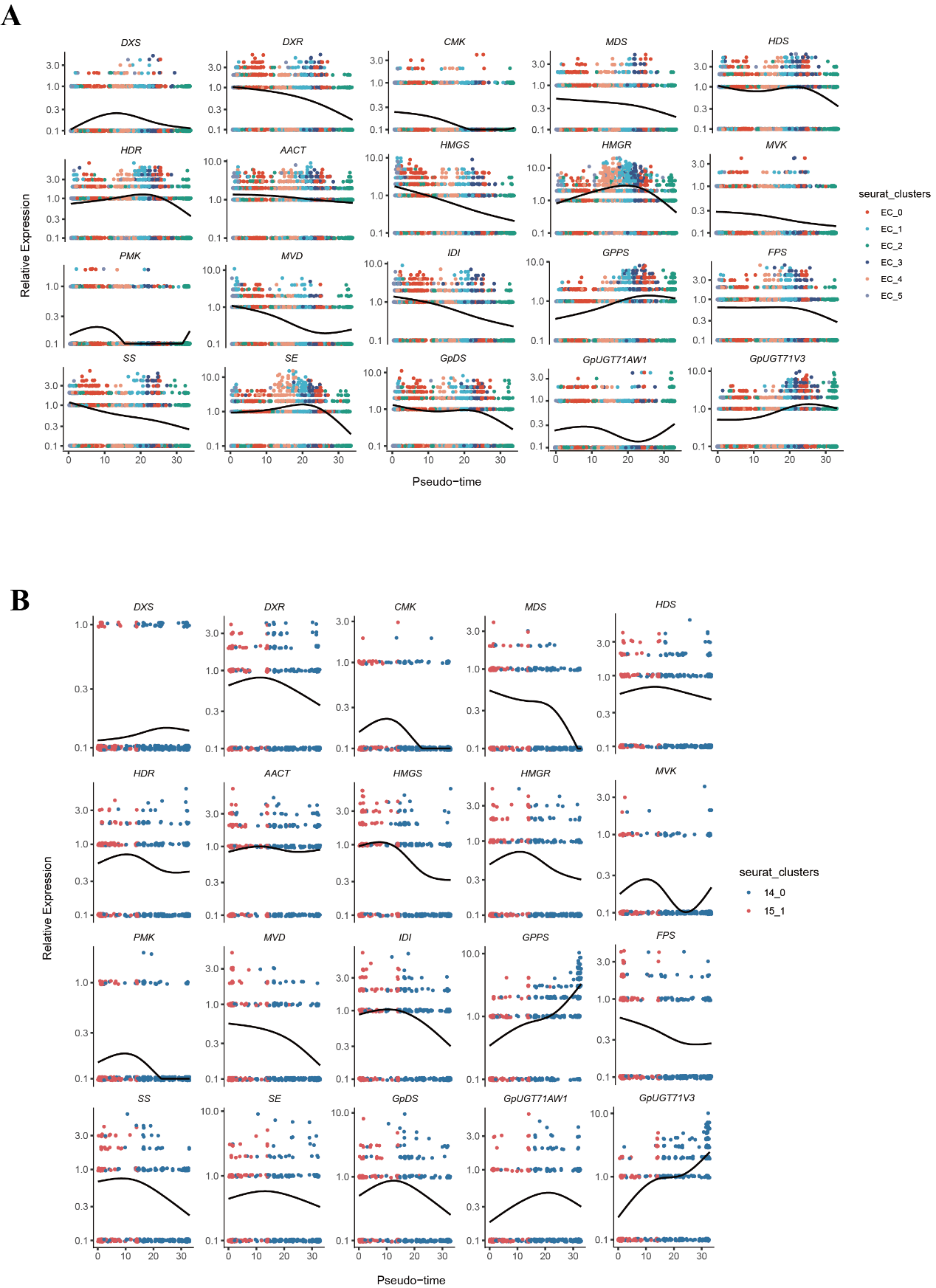


**Supplementary Figure 12. The dynamic expression patterns of genes involved in the gypenoside biosynthetic pathway during VC pseudotime progression.**
